# Mitochondria-targeted half-sandwich iridium(iii)-Cp*-arylimidazophenanthroline complexes as antiproliferative and bioimaging agents against triple negative breast cancer cells MDA-MB-468†

**DOI:** 10.1039/d2ra01036d

**Published:** 2022-04-19

**Authors:** Ashaparna Mondal, Shanooja Shanavas, Utsav Sen, Utpal Das, Nilmadhab Roy, Bipasha Bose, Priyankar Paira

**Affiliations:** Department of Chemistry, School of Advanced Sciences, Vellore Institute of Technology Vellore-632014 Tamilnadu India priyankar.paira@vit.ac.in; Department Stem Cells and Regenerative Medicine Centre, Institution Yenepoya Research Centre, Yenepoya University University Road, Derlakatte Mangalore 575018 Karnataka India bipasha.bose@gmail.com

## Abstract

To reduce the side effects of marketed cancer drugs against triple negative breast cancer cells we have reported mitochondria targeting half-sandwich iridium(iii)-Cp*-arylimidazophenanthroline complexes for MDA-MB-468 cell therapy and diagnosis. Out of five Ir(iii) complexes (IrL1–IrL5), [iridium(iii)-Cp*-2-(naphthalen-1-yl)-1*H*-imidazo[4,5-*f*][1,10]phenanthroline]PF_6_ (IrL1) has exhibited the best cytoselectivity against MDA-MB-468 cells compared to normal HaCaT cells along with excellent binding efficacy with DNA as well as serum albumin. The subcellular localization study of the complex revealed the localization of the compound in cytoplasm thereby pointing to a possible mitochondrial localization and consequent mitochondrial dysfunction *via* MMP alteration and ROS generation. Moreover, the IrL1 complex facilitated a substantial G_1_ phase cell-cycle arrest of MDA-MB-468 cells at the highest tested concentration of 5 μM. The study verdicts support the prospective therapeutic potential of the IrL1 complex in the treatment and eradication of triple negative breast cancer cells. These results validate that these types of scaffolds will be fairly able to exert great potential for tumor diagnosis as well as therapy in the near future.

## Abstract 2

Mitochondria targeting half-sandwich Iridium(iii)-Cp*-arylimidazophenanthroline complexes have been developed for MDA-MB-468 cell therapy and diagnosis.

## Graphical abstract

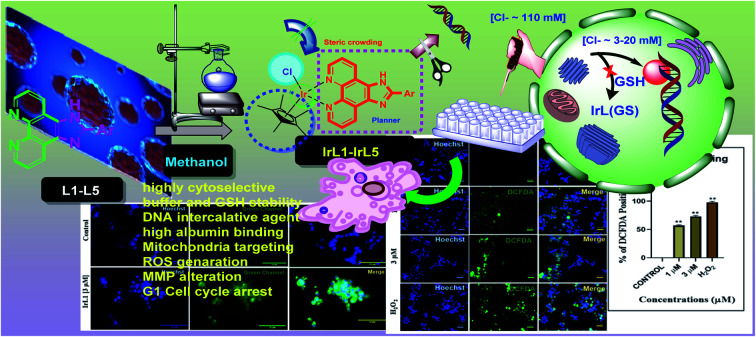

## Introduction

Nowadays, breast cancer prevails over most of the deadly life threats towards women from all over the world. 11.7% of new cases of cancer diagnosed in 2020 were female breast cancer which is the highest number of cases among all types of cancer reported.^1^ This disease is heterogenous at the molecular level but, as a consequence of diligent research over past decades, chances of healing have been increased by 70–80% in breast cancer patients when the cancer is non-metastatic and treated in the early stage. Over the years of research a few categorisations of tumours were refined based on the alterations at the molecular level.^2^ Current clinical practices involve an alternate classification of 5 types that includes triple negative breast cancer (TNBC) without expression of ER, PR or HER2. TNBC is adenoid cystic and metaplastic in nature with a poor prognosis profile.^2^ A thorough investigation of medical literature reveals that the term, “triple negative breast cancer”, was first mentioned in 2005 by Brenton and coworkers.^3^ However, in the present situation 12–17% of all reported breast cancer cases have been recognised under this class.

**Fig. 1:**
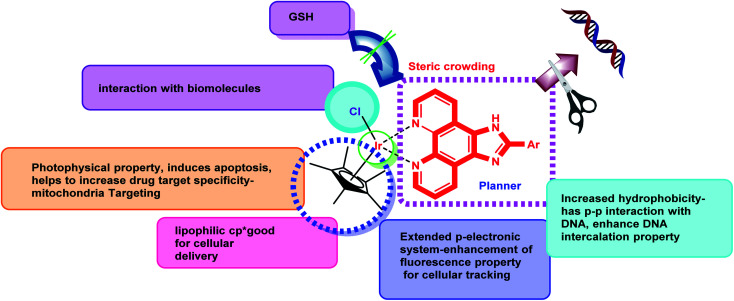
Design of half-sandwich iridium(iii)-Cp*-arylimidazophenanthroline complexes.

It has been challenging to create an effective treatment regime for TNBC patients as this aggressive subtype does not respond to the HER2 targeting drugs or hormonal therapy. Consequently, chemotherapy remains as the main systemic treatment option even though TNBC develops resistance easily to existing targeted medicines like trastuzumab. Potential molecular targets for TNBC may include EGFR (a surface receptor), PARP1 (poly ADP-ribose polymerase 1) and DNA. The phenotypic similarity of TNBC to BRCA-1 associated malignancy helped researchers to develop few targeted cytotoxic agents currently which may lead to a new horizon of TNBC therapeutics. In 2020 FDA approved trodelvy (sacituzumab govitecan-hziy) which is a topoisomerase inhibitor conjugate antibody directed to Trop-2 receptor for metastatic TNBC and for the first-time improved progression of overall survival was witnessed. Triple negative breast cancer caused by BRCA1 and BRCA2 mutation can be a potential target of DNA damaging chemotherapeutics and a number of clinical data leads to the suggestion that involving platinum-based chemotherapeutics may be use in standard treatment regime of early stage as well as advanced TNBC.^4^ As reported in earlier scientific research, platinum salts like cisplatin, carboplatin or oxaliplatin are able to initiate a platinum–DNA adduct formation followed by DNA damage in cancer cells leading towards apoptosis of the targeted cancer cells. BRCA1 deficient breast cancer showed sensitivity towards platinum and gemcitabine neoadjuvant treatment but with poor chance of progression free survival.^5^ A clinical study, reported by von Minckwitz in 2013 involving 315 early stage TNBC patients, let out the fact that a neoadjuvant treatment regime comprising doxorubicin and carboplatin achieved 59% pathologic complete response rate.^6^ However, a combination therapy of gemcitabine/carboplatin given to advanced metastatic TNBC patients in a trial scored 34% objective response rate and progression free survival of 5.1 months.^7^ Although cisplatin and its analogical platinum complexes showed slight prospect in TNBC treatment they have exerted some major drawbacks like toxicity, chemoresistance and narrow activity window.^8^ This fact motivated broader area of investigation on efficacy of other metal complexes to target metastasis and defective cell proliferation. A handful of gold, iron, copper, ruthenium, rhodium and iridium complexes happened to be synthesized and tasted against breast cancer cell lines *in vitro* or *in vivo* and found to manifest excellent outcomes.^9^ Of late, Ir(iii) based metal complexes have been fascinating the mind of the investigators for being reconnoitered as highly active anticancer agents with their excellent photoluminescence property possessing high photostabilities, long-lived excited triplet states after quick singlet to triplet intersystem crossing (ISC), larger Stokes' shift associated with high quantum yields.^10,11^ Furthermore, the higher oxidation state of iridium metal, ability to display a wide range of ligand substitution, considerable redox properties, flexible structural features and stability towards cancer cell environments has enabled them to be used in preparing anticancer metal complexes.^10,11^ Instead, bioactivities of metal complexes are also reliant on the structure of the ligands. Arylimidazophenthroline compounds are well known as pertinent probes of DNA structure having the capability of disentangling the double stranded DNA through strong intercalative interaction.^12^ Encouraged by our previous work with ruthenium and arylimidazophenthroline herein we have intended to design five iridium(iii)-Cp* N^N metal complexes with planar and π-extended arylimidazophenanthroline moiety as ligand having strong metal binding capacity (Fig. 1).^13–18^ and evaluated their antiproliferative activity against MDA-MB-468 TNBC cells that are EGFR +ve, TGF alpha +ve, high in Ki67. These complexes exhibited dual properties like (i) killing of cancer cells *via* DNA damage (ii) mitochondrial dysfunction by ROS production.^19^ The intrinsic phosphorescence property of these Ir(iii) complexes are also helpful for cellular imaging and tracking of drug accumulation in subcellular organelles.^20^

## Results and discussion

### Chemistry

#### Synthesis and characterization

**Scheme 1:**
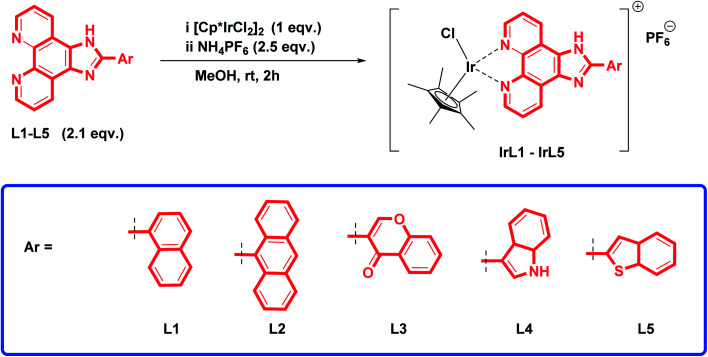
Synthesis of (η^5^-Cp*)iridium(iii)-imidazophenanthroline complexes.

A series of imidazo[4,5-*f*][1,10]phenanthroline ligands (L1–L5) was prepared by treating an equimolar mixture of 1,10-phenanthroline-5,6-dione and different aromatic carboxaldehydes (1–5) in the presence of ammonium acetate and glacial acetic acid, following the same procedure as mentioned in our previous communication.^21^ Further to prepare Ir(iii)-Cp*-imidazophenanthroline complexes (IrL1–IrL5), [(C_5_(CH_3_)_5_IrCl_2_)]_2_ was added to the prepared ligands (L1–L5) in a 1 : 2 ratio in methanol and stirred at room temperature for 2 h. After a change in colour from light yellow to orange, 2.5 equivalents of NH_4_PF_6_ were added and stirred again for 90 min (Scheme 1). The complexes [Cp*Ir(L1–L5)Cl]PF_6_ labelled as IrL1–IrL5 were obtained in good yield (92–95%). The structures of all the complexes (IrL1–IrL5) were analysed *via*^1^H, ^13^C, ^19^F and ^31^P NMR, and mass spectroscopy. The complex IrL1 displayed a characteristic singlet peak at 1.74 ppm, corresponding to the five methyl groups of pentamethylcyclopentadiene. The protons of complex IrL1 experienced a considerable downfield effect upon attachment to the iridium Cp* precursor. In the ^13^C NMR spectrum, the ligand carbons appeared at around *δ* 125.8–153.2 ppm. The aliphatic CH_3_ carbons peaks were observed at *δ* 8.7 ppm and aromatic CH carbons peaks were observed at *δ* 89.6 ppm. In the ^19^F NMR spectrum, characteristic peaks of six fluorines appeared at *δ* −69.2 and −71.08 ppm. The characteristic septate of phosphorous was observed in the range of *δ* −135.41 to −157.37 ppm in the ^31^P NMR spectrum. The ESI-MS peak at *m*/*z*: 709.17 [M]^+^ and isotopic pattern of iridium confirmed the formation of complex IrL1. Similarly, clear differences in peak values in the NMR, FT-IR and ESI-MS between the other complexes (IrL2–IrL5) were observed.

#### Electronic absorption (UV-visible) and fluorescence study

**Fig. 2:**
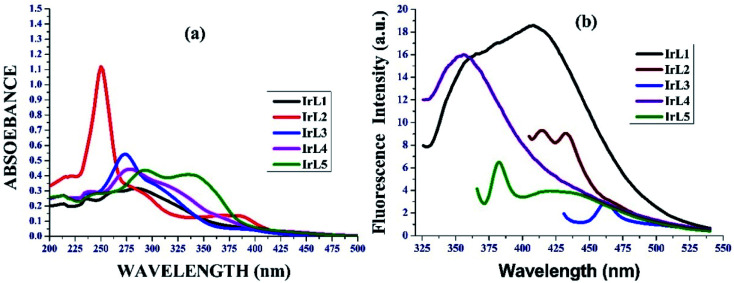
(a) UV-vis spectra (b) emission spectra of IrL1–IrL5 in DMSO–water (1 : 1) at RT.

The absorption and emission spectra of all the complexes (IrL1–IrL5) at 298 K were recorded in a DMSO–water (1 : 1) solvent system, as shown in Fig. 2. The photophysical data is summarized in Table 1. The characteristic intraligand (π–π*) transitions (N^N ligands) appeared at 250–350 nm and metal to ligand charge transfer (^1^MLCT) at 360–400 nm.^22,23^ Among the complexes, we observed the maximum absorption in the ^1^MLCT region for the benzothiazole derivative (IrL5). In the emission spectra, we observed the MLCT emission of all the complexes in the range of 350–525 nm (Fig. 2). Similar to the absorption spectra, the emission for the anthracene derivative is the most intense because of its strong π conjugation. Using the emission spectral data, the quantum yield of these complexes was calculated. These complexes didn't show remarkable quantum yield, complex IrL1 showed moderate quantum yield (0.002) though for the MLCT transition (Table 1).

**Table 1:** Photophysical characterization, solubility, lipophilicity and conductivity study of the complexes (IrL1–IrL5)

Samples	*λ*_max_ a (nm)	*λ*_f_ b (nm)	Stoke's shift	*ε* c (M^−1^ cm^−1^)	(*ϕ*_f_)^d^	Solubility^e^ (M)	log *P*^f^	*Λ*_M_ g (μs)	
								DMSO	10% DMSO
IrL1	284	409	125	15 800	0.002	0.0007	0.27 ± 0.07	8	22
IrL2	375	437	62	6850	—	0.0006	0.34 ± 0.07	6	27
IrL3	397	462	65	2200	—	0.0012	−0.23 ± 0.2	7	27
IrL4	278	360	82	2200	—	0.0009	0.02 ± 0.05	9	30
IrL5	336	380	44	2350	—	0.0007	0.29 ± 0.08	9	36
Quinine sulfate	350	452	102	—	0.57	—	—	—	—

a Absorption maxima.

b Maximum emission wavelength.

c Extinction coefficient.

d Quantum yield.

e DMSO-10% DMEM medium (1 : 99 v/v, comparable to cell media).

f *n*-Octanol/water partition coefficients.

g Conductance in DMSO and 10% aq. DMSO (IrL1–IrL5; 3 × 10^−5^ M).

#### Solubility, lipophilicity and conductivity study

Both hydrophilicity and lipophilicity studies were performed to determine the tumour-inhibiting potential of the metal complexes. These complexes were highly soluble in DMSO and moderately soluble in H_2_O, MeOH, EtOH and CH_3_CN. Furthermore, they were soluble in the range of 0.6–0.8 mg per mL of 10% DMSO in DMEM, 10 : 90 v/v (comparable to cell media) at 25 °C (Table 1). The lipophilicity of these complexes was determined by performing an *n*-octanol/water partition coefficient (log *P*_o/w_, where *P*_o/w_ = the octanol/water partition coefficient) study using the shake flask method (Table 1).^24^ The experimental log *P*_o/w_ values of these complexes were determined to be in the range of 0.27–0.34 (Table 1). Complex IrL2 exhibited the highest log *P*_o/w_ due to the hydrophobic nature of its anthracene group. The lowest log *P*_o/w_ value was observed for compound IrL3 because it's hydrophilic chromone group. The iridium complexes IrL1–IrL5 exhibited molar conductance values in the range of ∼7–9 S m^2^ M^−1^ in pure DMSO. Furthermore, their molar conductance increased in 10% DMSO (∼22–36 S m^2^ M^−1^, Table 1), suggesting their 1 : 1 and 1 : 2 electrolytic nature in pure DMSO and 10% DMSO, respectively.^25–27^ This change in the electrolytic behaviour of the complexes from 1 : 1 to 1 : 2 can be attributed to the dissociation of the Ir–Cl bond and subsequent aquation of the complexes.

#### Stability study of the complexes by UV-vis spectroscopy

**Fig. 3:**
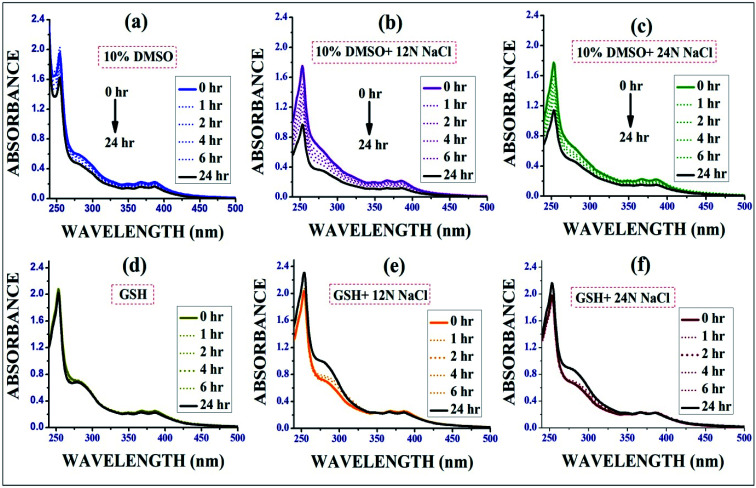
Stability study of IrL1 in (a) 10% DMSO media, (d) aqueous GSH media and in presence of 12N and 24N NaCl in each solution (b), (c), (e) and (f).

The stability studies of complex IrL1 were conducted in six different solvents, *i.e.* aqueous DMSO (H_2_O : DMSO = 9 : 1) and aqueous GSH medium (Fig. 3) respectively in presence or absence of different concentrations of NaCl. It is essential that the complexes remain stable in the biological environments of cells, and thus the stability studies were performed. The obvious change in absorbance (∼15–30% decrease after 24 h) with time in aqueous DMSO clearly revealed the moderate dissociation of the –Cl ligand from the Ir(iii) complexes followed by aqua complex formation, which was also quantitatively determined based on the observed molar conductivity of the complexes (IrL1–IrL5) in aqueous DMSO and it favours DNA covalent binding as well. It has been reported that many cancer cells become resistant to various drugs by increasing their cellular glutathione level.^28^ Hence, to determine the effect of GSH on the reported complexes, a stability study was performed in the presence of excess (10 eq.) glutathione (GSH) *via* time-dependent UV spectroscopy. However, GSH didn't have much impact on the stability of metal complex and hence resistivity may not be induced against this complex in cancer cells containing high level of GSH.

#### Cyclic voltammetry

**Fig. 4:**
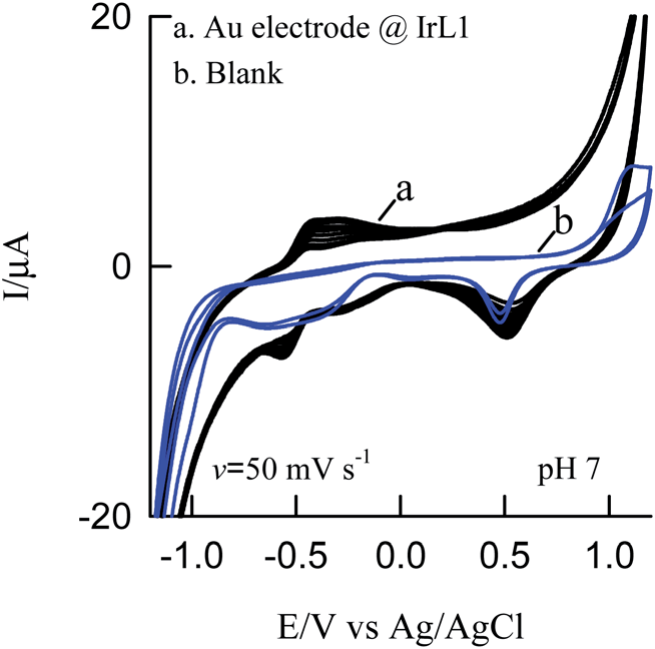
Cyclic voltammetry response of (a) Au modified IrL1 and (b) bare Au electrode in PBS buffer.

The CV response of ten-continuous cycle shows a well-defined reversible redox peak at *E*_0_ = −0.485 ± 0.005 V *versus* Ag/AgCl with a peak separation potential of Δ*E*_p_ value is 0.121 ± 0.005 (Δ*E*_p_ = *E*_pa_ − *E*_pc_). Peak current (*i*_pa_ and *i*_pc_) of the redox peak was found to increasing linearly which ensures the high stability and co-ordination of the metal–ligand complex (Fig. 4).

### Biology

#### *In vitro* cytotoxicity study

The *in vitro* cytotoxicity of complexes IrL1–IrL5 and cisplatin were investigated using the typical 3-(4,5-dimethylthiazol-2-yl)-2,5-diphenyltetrazolium bromide (MTT) assay protocol against a panel of triple negative cancer cell line MDA-MB-468. The cells were initially incubated with the all test compounds at concentrations ranging from 0.5–50 μM for 48 h *in vitro*.

**Fig. 5:**
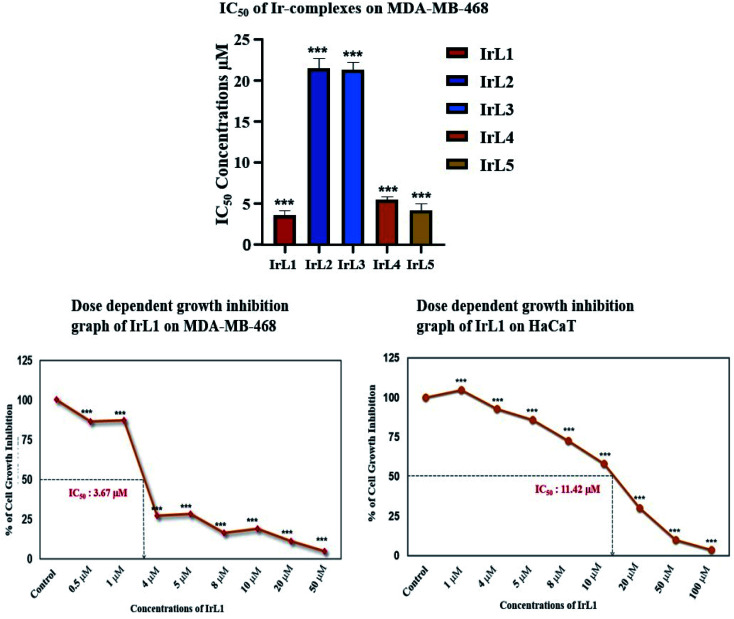
Cytotoxicity study of IrL1 against TNBC and normal cells.

All the complexes exhibited significant cytotoxicity with IC_50_ values of 3.673 μM, 20.35 μM, 20.27 μM, 5.202 μM, 4.412 μM for IrL1, IrL2, IrL3, IrL4 and IrL5 respectively (Fig. 4). The dose dependent cytotoxic effects of these complexes were evident on MDA-MB-468 with IrL1 exhibiting the most potent effects thereby exhibiting the lowest IC_50_ value of 3.67 μM (Fig. 5). Consequently, the IrL1 complex doses of 1 μM, 3 μM and 5 μM were selected for further experimental analysis. Furthermore, the possible cytotoxic effects of IrL1 on normal cell line were also examined on the immortalized human keratinocyte cell line HaCaT. The IC_50_ value of IrL1 on HaCaT is found to be 11.42 μM, which is significantly ∼3 times higher than the IC_50_ value of the complex on the breast cancer cell line MDA-MB-468, suggesting the low cytotoxic effects of the same on normal cells and safety implications to use the compound for selective therapeutics at lower concentrations (Fig. 4).

### DNA binding studies

#### UV absorption method

**Fig. 6:**
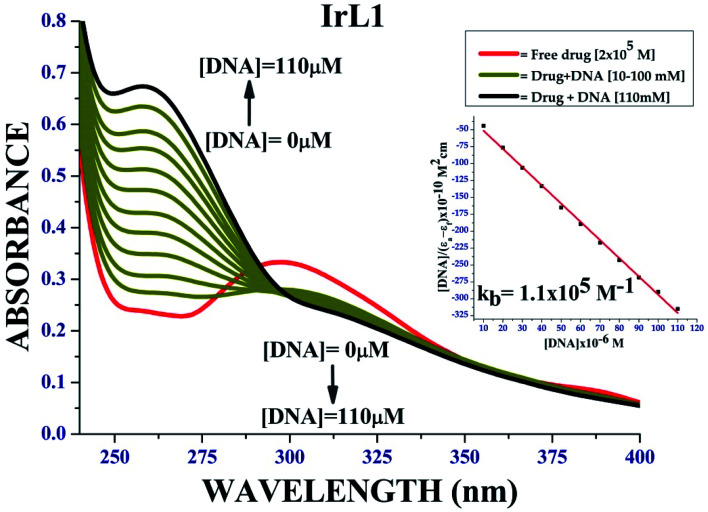
DNA binding plot of complex IrL1; inset: [DNA]/(*ε*_a_ − *ε*_f_) *vs.* [DNA] linear plots of IrL1.

To design effective chemotherapeutic drugs, it is essential to explore the interactions of metal complexes with DNA. Complex IrL1 displayed strong absorption band at 297 nm. DNA base pairs such as purine (adenine and guanine) and pyrimidine (cytosine and thymine) analogues are responsible for electronic transitions. Upon the addition of CT-DNA in increasing concentration from 5 μM to 60 μM, we observed a hyperchromic shift in π–π* region and hypochromic shift at the MLCT region resulting in the appearance of an isosbestic point, which indicates the prevalence of covalent interaction of the complex with DNA together with the intercalative mode of interaction (Fig. 6).

#### EtBr quenching study

**Fig. 7:**
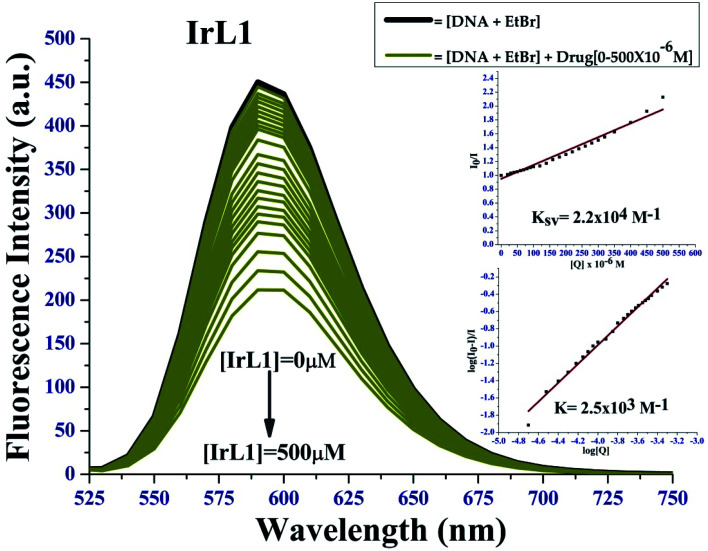
Fluorescence quenching plot of complex IrL1; inset: *I*_0_/*I vs.* [complex] linear plots of IrL1.

The competitive binding of compounds IrL1 to CT-DNA was studied *via* fluorescence spectroscopy using ethidium bromide (EtBr) as the fluorophore. We clearly observed a gradual decrease in the fluorescence intensity of the EtBr bound DNA in the presence of the complexes since they displaced EtBr from DNA, and consequently got bound between the base pairs of the DNA, suggesting the intercalative binding mode of action, as observed in Fig. 7. Intrinsic binding constant (*K*_b_), Stern–Volmer quenching constant (*K*_sv_) and apparent binding constant (*K*_app_) were highlighted in Table 2.

**Table 2:** Binding parameters for complex IrL1 with CT-DNA

Complex	*λ*_max_ (nm)	Change in absorbance	Δ*ε*^a^ (%)	*K*_b_ b (M^−1^)	*K*_sv_ c (M^−1^)	*K*_app_ d (M^−1^)
IrL1	250	Hyperchromism	60	—	—	—
	305	Hypochromism	25	0.11 × 10^6^	2.2 × 10^4^	2.5 × 10^3^

a % change in hypochromism or hyperchromism.

b *K*_b_, intrinsic DNA binding constant from UV-visible absorption titration.

c *K*_sv_, Stern–Volmer quenching constant.

d *K*_app_, apparent DNA binding constant from competitive displacement.

The higher value of binding constant and lower values of Stern–Volmer's quenching constant and apparent binding constant is evidence of the fact that IrL1 complex interacts with DNA *via* covalent bond and not *via* intercalation mode.

#### BSA binding study

**Fig. 8:**
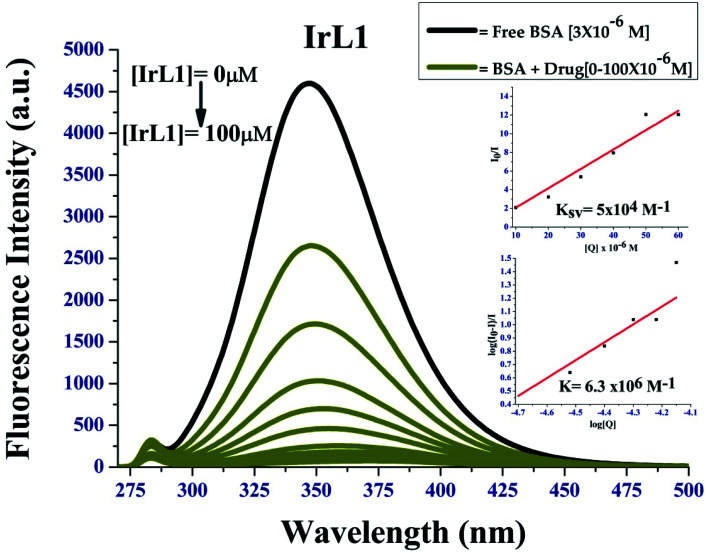
Fluorescence quenching of BSA by IrL1; inset: *I*_0_/*I vs.* [complex] linear plots for stern Volmer's constant and Scatchard plot of log([*I*_0_ − *I*]/*I*) *vs.* log[complex] for BSA.

Upon excitation at 295 nm, the emission intensity of BSA at *λ*_em_ = 350 nm decreased gradually on increasing the complex concentration, which confirmed that the interaction between complex IrL1 with BSA had occurred, as observed in Fig. 8. The Stern–Volmer quenching constant of these complexes with BSA (*K*_BSA_) was calculated using the Stern–Volmer equation and the corresponding Stern–Volmer plots (Fig. 8, inset). The binding affinity (*K*) of the complexes was calculated from Scatchard plot analysis (Fig. 8, inset).

The complexes showed strong binding propensity with BSA, which is required for the transport of protein-bound complexes in biological systems. *K*_BSA_ for complex IrL1 was found to be 5 × 10^4^ M^−1^ the *K* value was 6.3 × 10^6^ M^−1^. The value of bimolecular quenching constant (*k*_q_) calculated from *K*_SV_ and *τ*^0^ (1 × 10^−8^ s) was observed to be 5 × 10^12^ M^−1^ S^−1^. These values are higher than the maximum possible value for dynamic quenching (2.0 × 10^10^ L mol^−1^ s^−1^),^29,30^ suggesting the involvement of static quenching mechanism by the present iridium(iii) complexes. The non-linearity of the Stern–Volmer plot of IrL1 is due to the formation of a ground state complex between the Ir(iii) complex and BSA.

#### Co-localization study

**Fig. 9:**
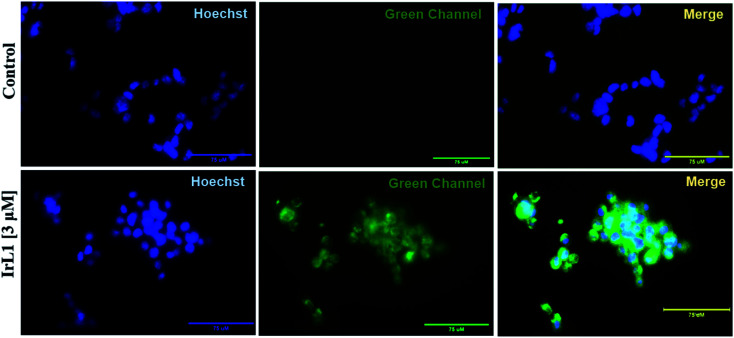
Representative image of the cytoplasmically localized IrL1 complex in MDA-MB-468 cells, co-stained with Hoechst. Scale bar 75 μM.

To identify the subcellular localization of the IrL1 complex, the cells were treated with the complex and stained with Hoechst and further explored using fluorescence microscopy. The complex exhibited cytoplasmic localization at 3 μM concentration with a green fluorescence emission (Fig. 9). Mitochondria are one of the key targets for several anticancer drugs. Consequently, further experimental validation involved mitochondrial membrane potential (MMP) analysis by JC-1 staining and detection of ROS generation by DCFDA staining.

#### Mitochondrial membrane dysfunction study

The IrL1 complex demonstrated mitochondrial depolarization of MDA-MB-468 cells in a dose dependent manner. The cytoplasmic localization of the IrL1 complex as evidenced by sub-cellular localization study impelled us to explore the effect of the complex on cytoplasmic mitochondria. Consequently, the alterations in mitochondrial membrane potential (MMP, Δ*Ψ*_m_) and associated mitochondrial dysfunction was demonstrated by JC-1, a cationic carbocyanine dye which exhibits potential-dependent accumulation in mitochondria. The cells treated with the mitochondrial uncoupler CCCP (carbonyl cyanide *m*-chlorophenylhydrazone), which mediates the dissipation of mitochondrial membrane potential, served as positive control for the detection of mitochondrial dysfunction.

**Fig. 10:**
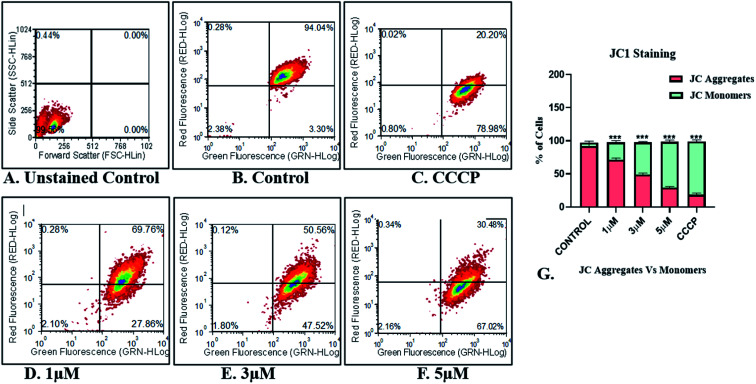
Image representing the flow cytometric quantification of JC-1-stained MDA-MB-468 breast cancer cells for mitochondrial membrane potential assessment (A) unstained control for gating purposes (B) untreated control (C) positive control (CCCP treated cells) (D) 1 μM treatment (E) 3 μM treatment (F) 5 μM treatment (G) graph representing the percentage of JC-1 monomers/aggregates.

The flow cytometric quantification of JC-1-stained cells revealed a normal mitochondrial function in control cells devoid of any treatment exhibiting ∼94% JC-1 aggregates (red+ green fluorescing healthy mitochondria). Impaired mitochondrial function was observed in only 3.30% of control cells with JC-1 monomers indicating green fluorescence (Fig. 10B). Unstained control represented in Fig. 10A has been used for gating purposes. Conversely, the positive control with CCCP treatment, displayed a high percentage of cells (∼79%) with damaged mitochondria indicating the presence of green fluorescing JC-1 monomers against ∼20% cells retaining healthy mitochondria with red + green fluorescing JC-1 aggregates (Fig. 10C).

Treatment of the MDA-MB-468 cells with IrL1 has ensued a dose dependent increase in JC-1 monomer expressing/green fluorescing cells indicating damaged mitochondria (Fig. 10D–F). IrL1 complex at 1 μM concentration resulted in damaged mitochondria in 27.86% cells (Fig. 10D). However, the higher concentrations of the IrL1 complex at 3 and 5 μM induced alterations of mitochondrial membrane potential and associated damage in high percentage ∼47.52% and 67.02% respectively in MDA-MB-468 breast cancer cells (Fig. 10E and F). The proportion of green fluorescing JC-1 monomers induced by high concentrations of the complex at 3 and 5 μM thereby indicates alteration in the MMP and mitochondrial depolarization and associated mitochondrial dysfunction leading to a dose dependent possible cell death meditated by the IrL1 complex comparable to the CCCP treated positive control cells (Fig. 10C).

#### ROS generation studies

The IrL1 complex exhibited a dose dependent reactive oxygen species (ROS) generation in the MDA-MB-468 breast cancer cells. The MDA-MB-468 breast cancer cells exhibited ROS generation suggestive of cellular stress leading towards cell death at elevated ROS levels.

**Fig. 11:**
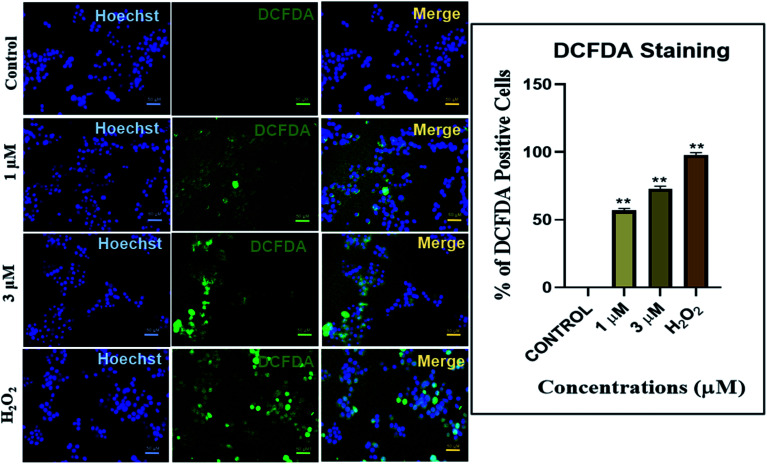
Reactive oxygen species (ROS) assessment as a measure of cellular damage after treating the MDA-MB468 breast cancer cells with various concentrations of IrL1 compound and the positive control H_2_O_2_.

Our results indicated a dose dependent increase in ROS production in the MDA-MB-468 cells treated with IrL1 concentrations 1 μM and 3 μM. The extent of ROS production is indicated by the 2′,7′-dichlorofluorescein (DCF) formed by the deacetylation and subsequent oxidation of 2′,7′-dichlorofluorescin diacetate (DCFDA) through cellular esterases and ROS. 1 μM concentration of the complex has resulted in 55.01% DCF positive cells which has increased up to 70.67% DCF positive cells at 3 μM concentrations. ROS production was absent in untreated control. However, in the positive control, treated with H_2_O_2_, ROS production was high which is ∼99% (Fig. 11).

#### Cell cycle analysis

**Fig. 12:**
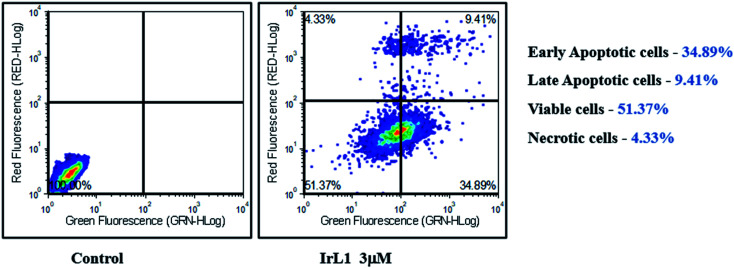
Analysis of apoptotic MDA-MB-468 cells treated with IrL1 complex by Annexin V-FITC/PI assay. The 3 μM concentration of the drug (IC-50) has resulted in 34.89% early apoptotic cells, 9.41% of late apoptotic cells and 4.33% necrotic cells. The viable cell population was found to be 51.37%.

The IrL1 complex exerted a G_0_/G_1_ cell cycle arrest at the highest tested concentration of 5 μM. The effects of various concentrations of IrL1 complex on the cell cycle of breast cancer cell line MDA-MB-468 cells were further explored by cell cycle analysis. The untreated control breast cancer cells demonstrated high S phase (∼35.16%), followed by a G_2_/M phase with ∼33.18% cells, and 31.66% of G_0_/G_1_ cells (Fig. 12). Conversely, the treatment of IrL1 complex resulted in a decrease of G_2_/M phase cells upto ∼18.37% and a decrease of S phase cells upto ∼28.32% with the highest concentration of the complex *i.e.*, 5 μM. Remarkably, 5 μm concentration considerably increased G_0_/G_1_ phase cells (53.32%), indicative of a substantial G_1_ arrest of MDA-MB-468 breast cancer cells mediated by the IrL1 complex.

#### Annexin FITC/PI assay

**Fig. 13:**
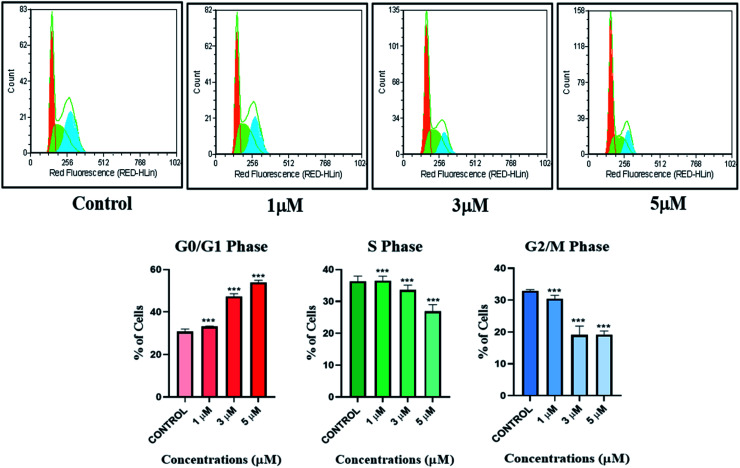
Cell cycle analysis of untreated and IrL1 treated (1 μM, 3 and 5 μM) MDA-MB-468 cells and representative graph of the cell cycle phases up on treatment with IrL1 complex. IrL1 mediates G_1_ arrest of MDA-MB-468 cells at 5 μM concentration. The *p* value of <0.0001 (***) was considered statistically significant. Error bar represents the ±standard error of mean (SEM).

The Annexin FITC/PI assay has demonstrated significant apoptotic initiation of IrL1 treated MDA-MB-468 breast cancer cells. At 3 μM concentration of the complex, ∼34.89% of cells were found to be displaying early apoptotic phase and ∼9.49% cells exhibited late apoptotic phase (Fig. 13). The viable cell population constituted ∼51.37% which supports the IC_50_ concentration of 3.673 μM demonstrated by IrL1 on MDA-MB-468 cells thereby corroborating the IC_50_ data at this particular concentration.

## Experimental section

### Materials and methods

In all the experiments, the reagents and solvents used were of the highest grade and best commercial quality. All organic solvents used throughout the chemical synthesis and chromatography procedures were of analytical grade and used without further purification as received from E. Merck (India). Pentamethylcyclopentadienyl iridium(iii) chloride dimer, 1,10-phenanthroline-5,6-dione, α-naphthaldehyde, 9-anthraldehyde, chromone-3-carboxaldehyde, indole-3-carboxaldehyde and benzothiazole-2-carboxaldehyde were procured from Sigma Aldrich Chemical Ltd, Merck and Spectrochem. Thin layer chromatography was performed on pre-coated silica gel 60 F_254_ aluminium sheets (E. Merck, Germany) and the solvent system was an ethyl–acetate–methanol mixture.

^1^H NMR, ^13^C NMR, ^19^F NMR and ^31^P NMR spectra were recorded on a 400 MHz Advance Bruker DPX spectrometer with tetramethylsilane (TMS) as the internal standard. The chemical shifts were reported in ppm units. Abbreviations are as follows: s, singlet; d, doublet; dd, double doublet; t, triplet; and m, multiplet. The melting points of the complexes were measured on an Elchem Microprocessor-based DT apparatus using an open capillary tube. The mass spectra of the synthesized compounds were recorded on a Shimadzu ESI-mass spectrometer having a 4000 triple quadrupole MS, using methanol as the solvent. UV-visible spectra were recorded on a JASCO V-730 spectrometer using a 1 cm quartz cell and fluorescence spectra on Hitachi F7000 fluorescence spectrophotometer equipped with a xenon lamp. A PerkinElmer instrument was used for the elemental analysis. The conductivity and viscosity study were performed using a conductivity-TDS meter-307 and Ostwald viscometer, respectively.

For the cytotoxicity (MTT) assay and imaging study, an Elisa reader, 96-well plate and Olympus CX41 fluorescence microscope were used. Bovine serum albumin (BSA) was purchased from Sigma Aldrich Chemical Limited. The MDA-MB-468 and HaCaT cell lines were purchased from NCCS, Pune.

### Synthetic procedure

Synthesis of [Ir(iii)-Cp*-(arylimidazophenanthroline)Cl]PF_6_ complexes [IrL1–IrL5]: 30 mg (0.037 mmole, 1 eq.) of pentamethylcyclopentadienyl iridium dichloride dimer, [(C_5_(CH_3_)_5_IrCl_2_)]_2_, was dissolved in about 10 ml of methanol in a 50 ml round bottomed flask and was stirred continuously for 5–10 min to dissolve the reactant. To the completely dissolved solution, 2.1 equivalents of the previously synthesized ligands (L1–L5) were added and stirred at room temperature. After 90 min, 2.5 equivalents of ammonium hexafluorophosphate (NH_4_PF_6_) (13.3 mg, 0.082 mmol) was added as ligand exchange salt in order to increase crystallinity and hence, purity of the product and again the reaction mixture was stirred for 90 min more at room temperature. The progress of reaction was confirmed by TLC. After complete conversion of the starting materials to the desired product, the solvent was evaporated under reduced pressure. The crude product was washed with hexane and further recrystallized from diethyl ether/methanol (1 : 1) solvent system. Finally, the complexes (IrL1–IrL5) were obtained as light brown crystals with high yield (90–92%).

### Characterization data for complexes (IrL1–IrL5)

#### [Iridium(iii)-Cp*-2-(naphthalen-1-yl)-1*H*-imidazo[4,5-*f*][1,10]phenanthroline]PF_6_ (IrL1)

60 mg (0.0702 mmol, 95%); Mr (C_33_H_29_ClF_6_IrN_4_P) = 854.25 g mol^−1^; anal. calcd for C_33_H_29_ClF_6_IrN_4_P: C 46.40, H 3.42, N 6.56; found: C 46.12; H 3.09; N 6.23; *R*_f_ (100% methanol): 0.2; mp: >200 °C; IR (cm^−1^): *ν* 3612, 3350, 3100, 1605, 1403, 1363, 1089, 836, 743, 566; ^1^H NMR (DMSO-*d*_6_, 400 MHz): *δ* 1.74 (s, 15H, Cp* aliphatic-CH_3_); 7.63–7.78 (m, 3H, ligand ArH); 8.08 (d, 1H, *J* = 7.6 Hz ligand ArH); 8.16–8.22 (dd, 2H, *J* = 8.0 Hz, ligand ArH); 8.27 (m, 2H, ligand ArH); 8.44 (m, 2H, ligand ArH); 9.08 (d, 1H, *J* = 8.2 Hz, ligand ArH); 9.34 (d, 2H, *J* = 5.2 Hz, ligand ArH); 9.55 (d, 1H, *J* = 10.8 Hz, ligand ArH); ^13^C NMR (DMSO-*d*_6_, 100 MHz): *δ* 8.7 (Cp*-aliphatic Me), 89.63–90 (Cp*-aromatic carbon), 125.81 (2C, ArC), 127.07 (2C, ArC), 127.95 (4C, ArC), 128.96 (2C, ArC), 130.8 (2C, ArC), 131.2 (2C, ArC), 133.5 (2C, ArC), 134.08 (2C, ArC), 150.58 (2C, ArC), 153.24 (2C, ArC); ^19^F NMR (DMSO-*d*_6_, 376 MHz): *δ* −71.07 to −69.18 (6F, PF_6_); ^31^P NMR (DMSO-*d*_6_, 162 MHz): *δ* −152.98 to −135.43 (PF_6_); ESI-MS (MeOH): *m*/*z* = 709.17 [M]^+^.

#### [Iridium(iii)-Cp*-2-(anthracen-1-yl)-1*H*-imidazo[4,5-*f*][1,10]phenanthroline]PF_6_ (IrL2)

63 mg (0.069 mmol, 93%); Mr (C_37_H_31_ClF_6_IrN_4_P) = 904.31 g mol^−1^; anal. calcd for C_37_H_31_ClF_6_IrN_4_P: C 49.14, H 3.46, N 6.20; found: C 49.35; H 3.63; N 6.02; *R*_f_ (100% methanol): 0.2; mp: >200 °C; IR (cm^−1^): *ν* 3626, 3056, 1613, 1450, 1136, 835, 741, 556; ^1^H NMR (DMSO-*d*_6_, 400 MHz): *δ* 1.76 (s, 15H, Cp* aliphatic-CH_3_); 7.53 (t, 2H, *J* = 8.0 Hz, ligand ArH); 7.62 (t, 2H, *J* = 8.0 Hz ligand ArH); 7.75 (brs, 2H, ligand ArH); 8.28 (d, 4H, *J* = 8.0 Hz ligand ArH); 8.96 (s, 1H, ligand ArH); 9.17 (d, 1H, *J* = 8.0 Hz, ligand ArH); 9.31 (d, 1H, *J* = 8.0 Hz, ligand ArH); 9.37 (s, 1H, ligand ArH); ^13^C NMR (DMSO-*d*_6_, 100 MHz): *δ* 8.7 (Cp*-aliphatic Me), 89.68 (Cp*-aromatic carbon), 124.75 (3C, ArC), 125.75 (4C, ArC), 126.34 (4C, ArC), 127.85 (2C, ArC), 128.07 (3C, ArC), 129.16 (2C, ArC), 130.12 (3C, ArC), 131.1 (4C, ArC), 131.2 (4C, ArC), 133.3 (1C, ArC); 144.6 (2C, ArC); 150.5 (2C, ArC); 151.2 (1C, ArC); ^19^F NMR (DMSO-*d*_6_, 376 MHz): *δ* −71.07 to −69.18 (6F, PF_6_); ^31^P NMR (DMSO-*d*_6_, 162 MHz): *δ* −152.98 to −135.43 (PF_6_); ESI-MS (MeOH): *m*/*z* = 759.19 [M]^+^.

#### [Iridium(iii)-Cp*-3-(1*H*-imidazo[4,5-*f*][1,10]phenanthrolin-2-yl)-4*H*-chromen-4-one]PF_6_ (IrL3)

61 mg (0.07 mmol, 95%); Mr (C_32_H_27_ClF_6_IrN_4_P) = 872.23 g mol^−1^; anal. calcd for C_32_H_27_ClF_6_IrN_4_P: C 44.07, H 3.12, N 6.42; found: C 44.32; H 3.45; N 6.12; *R*_f_ (100% methanol): 0.2; mp: >200 °C; IR (cm^−1^): *ν* 3251, 1640, 1462, 1375, 1143, 1027, 836, 759, 555; ^1^H NMR (DMSO-*d*_6_, 400 MHz): *δ* 1.73 (s, 15H, Cp* aliphatic-CH_3_); 7.66 (t, 2H, *J* = 7.6 Hz, ligand ArH); 7.85 (d, 1H, *J* = 8.2 Hz, ligand ArH); 7.95 (t, 1H, *J* = 7.6 Hz, ligand ArH); 8.24–8.31 (m, 4H, ligand ArH); 9.32 (s, 2H, ligand ArH); 9.43 (s, 1H, ligand ArH); ^13^C NMR (DMSO-*d*_6_, 100 MHz): *δ* 8.6 (Cp*-aliphatic Me), 89.7 (Cp*-aromatic carbon), 92.7 (1C, ArC), 114.47 (1C, ArC), 119.05 (2C, ArC), 123.50 (1C, ArC), 125.59 (1C, ArC), 126.94 (1C, ArC), 127.81 (2C, ArC), 133.52 (1C, ArC), 135.49 (1C, ArC), 144.43 (2C, ArC), 147.01 (2C, ArC), 150.41 (2C, ArC), 155.82 (1C, ArC), 158.58 (1C, ArC), 174.75 (1C, ArC); ^19^F NMR (DMSO-*d*_6_, 376 MHz): *δ* −71.07 to −69.18 (6F, PF_6_); ^31^P NMR (DMSO-*d*_6_, 162 MHz): *δ* −152.98 to −135.43 (PF_6_); ESI-MS (MeOH): *m*/*z* = 727.15 [M]^+^.

#### [Iridium(iii)-Cp*-2-((3*aR*,7*aR*)-3*a*,7*a*-dihydro-1*H*-indol-3-yl)-1*H*-imidazo[4,5-*f*][1,10]phenanthroline]PF_6_ (IrL4)

57 mg (0.068 mmol, 92%); Mr (C_31_H_28_ClF_6_IrN_5_P) = 843.23 g mol^−1^; anal. calcd for C_31_H_28_ClF_6_IrN_5_P: C 44.16, H 3.35, N 8.31; found: C 44.31; H 3.59; N 8.67; *R*_f_ (100% methanol): 0.2; mp: >200 °C; IR (cm^−1^): *ν* 3112, 1635, 1448, 1393, 1090, 837, 727, 559; ^1^H NMR (DMSO-*d*_6_, 400 MHz): *δ* 1.73 (s, 15H, Cp* aliphatic-CH_3_); 7.54–7.57 (q, 1H, *J* = 2.8 Hz ligand ArH); 8.22–8.25 (q, 3H, *J* = 4.4 Hz ligand ArH); 8.58 (s, 1H, ligand ArH); 8.7 (s, 1H, ligand ArH); 9.29 (d, 3H, *J* = 5.2 Hz, ligand ArH); 9.4 (brs, 2H, ligand ArH); 11.84 (s, 1H, ligand NH); ^13^C NMR (DMSO-*d*_6_, 100 MHz): *δ* 8.62 (Cp*-aliphatic Me), 41.69 (1C, ArC), 89.64 (Cp*-aromatic carbon), 92.80 (3C, ArC), 106.21 (2C, ArC), 121.32 (1C, ArC), 123.24 (2C, ArC), 125.26 (1C, ArC), 127.02 (1C, ArC), 127.58 (2C, ArC), 133.02 (2C, ArC), 136.94 (1C, ArC), 143.85 (2C, ArC), 149.86 (2C, ArC), 151.67 (1C, ArC); ^19^F NMR (DMSO-*d*_6_, 376 MHz): *δ* −71.07 to −69.18 (6F, PF_6_); ^31^P NMR (DMSO-*d*_6_, 162 MHz): *δ* −152.98 to −135.43 (PF_6_); ESI-MS (MeOH): *m*/*z* = 698.17 [M]^+^.

#### [Iridium(iii)-Cp*-2-((3*aR*,7*aR*)-3*a*,7*a*-dihydrobenzo[*b*]thiophen-2-yl)-1*H*-imidazo[4,5-*f*][1,10]phenanthroline]PF_6_ (IrL5)

58 mg (0.068 mmol, 92%); Mr (C_31_H_27_ClF_6_IrN_4_PS) = 860.28 g mol^−1^; anal. calcd for C_31_H_27_ClF_6_IrN_4_PS: C 43.28, H 3.16, N 6.51; found: C 43.47; H 3.41; N 6.72; *R*_f_ (100% methanol): 0.2; mp: >200 °C; IR (cm^−1^): *ν* 3120, 2926, 1604, 1448, 1393, 1090, 837, 727, 559; ^1^H NMR (DMSO-*d*_6_, 400 MHz): *δ* 1.72 (s, 15H, Cp* aliphatic-CH_3_); 7.47 (s, 2H, ligand ArH); 8.01–8.08 (dt, 2H, *J* = 6.7 Hz ligand ArH); 8.23–8.26 (m, 2H, ligand ArH); 8.34 (s, 1H, ligand ArH); 9.23 (d, 2H, *J* = 8.0 Hz, ligand ArH); 9.3 (d, 2H, *J* = 5.2 Hz, ligand ArH); ^13^C NMR (DMSO-*d*_6_, 100 MHz): *δ* 8.67 (Cp*-aliphatic Me), 49.06 (1C, ArC), 89.67 (Cp*-aromatic carbon), 92.63 (1C, ArC), 123.22 (2C, ArC), 124.13 (1C, ArC), 125.11 (1C, ArC), 125.76 (1C, ArC),126.52 (1C, ArC), 127.96 (1C, ArC), 133.29 (2C, ArC), 139.98 (2C, ArC), 140.09 (2C, ArC), 144.56 (2C, ArC), 150.54 (2C, ArC); ^19^F NMR (DMSO-*d*_6_, 376 MHz): *δ* −71.07 to −69.18 (6F, PF_6_); ^31^P NMR (DMSO-*d*_6_, 162 MHz): *δ* −152.98 to −135.43 (PF_6_); ESI-MS (MeOH): *m*/*z* = 715.13 [M]^+^.

### Cytotoxicity studies

Cytotoxicity study of all the synthesized compounds against triple negative breast cancer cells MDA-MB-468 and immortalized human keratinocyte cell line HaCaT were done with a drug incubation period of 48 h by following standard procedure that were mentioned in ESI.† Preliminary studies were done to understand the mode of action of the complex to induce cytotoxicity using flow cytometric methods.

### Mitochondrial membrane dysfunction assay

Alterations in mitochondrial membrane potential (MMP, Δ*Ψ*_m_) and associated mitochondrial dysfunction was demonstrated by JC-1, a cationic carbocyanine dye. The cells were treated with the mitochondrial uncoupler CCCP (carbonyl cyanide *m*-chlorophenylhydrazone), which mediates the dissipation of mitochondrial membrane potential, served as positive control for the detection of mitochondrial dysfunction. The flow cytometric quantification of JC-1 stained cells revealed a normal mitochondrial function in control cells and the results obtained from the quantified JC-1 stained cells among IrL1 treated cells were compared with the same of the control.

### Imaging studies

Colocalization study and ROS generation studies were performed using live cell lines in their log phase.

### Statistical analysis

As the study had more than one group, one way ANOVA was used for statistical analysis. The *p* value <0.05 was considered as significant.

## Conclusions

Five Ir(iii)-imidazophenanthroline complexes were synthesized and characterized successfully. Our study has demonstrated the cytotoxic potential of IrL1 complex on MDA-MB-468 breast cancer cells in a dose dependent manner. The subcellular localization study of the complex revealed the localization of the compound in cytoplasm thereby pointing to a possible mitochondrial localization and consequent mitochondrial dysfunction. Subsequent analysis to demonstrate the alterations in MMP and ROS generation mediated by the IrL1 complex has revealed a significant increase in mitochondrial dysfunction and a resultant increase in ROS production suggestive of the mitochondrial targeting potential of the IrL1 complex. The Annexin V-FITC/PI assay has validated the cytotoxic potential, along with the IC_50_ dose of the complex on MDA-MB-468 breast cancer cells by initiating apoptotic pathway probably due to the cellular energetic stress triggered by elevated ROS levels. Furthermore, the IrL1 complex mediated a substantial G_1_ phase cell-cycle arrest of MDA-MB-468 cells at the highest tested concentration of 5 μM. The study findings support the prospective therapeutic potential of the IrL1 complex in the treatment and eradication of triple negative breast cancer cells.

## Conflicts of interest

There are no conflicts to declare.

## Supplementary Material

RA-012-D2RA01036D-s001
