# Supplementary material for: Mitochondria-targeted half-sandwich iridium(iii)-Cp*-arylimidazophenanthroline complexes as antiproliferative and bioimaging agents against triple negative breast cancer cells MDA-MB-468
Source: RSC Adv. 2022 Apr 19;12(19):11953–66. doi: 10.1039/d2ra01036d (PMC9016803; doi:10.1039/d2ra01036d)
Supplement: RA-012-D2RA01036D-s001 [file RA-012-D2RA01036D-s001.pdf]

Electronic Supplementary Material (ESI) for RSC Advances.
This journal is © The Royal Society of Chemistry 2022

Supporting Information

Mitochondria-targeted half-sandwich Iridium(III)-Cp*-arylimidazophenanthroline complexes as antiproliferative and bioimaging agent against triple negative breast cancer cells MDA-MB-468

Ashaparna Mondal,[a]† Shanooja Shanavas,[b]† Utsav Sen,[b] Utpal Das,[a] Nilmadhab Roy, [a] Bipasha Bose,[b]* Priyankar Paira[a]*

[a]*Department of Chemistry, School of advanced sciences, Vellore Institute of Technology Vellore-632014, Tamilnadu, India, E-mail: priyankar.paira@vit.ac.in*

[b]*Department Stem Cells and Regenerative Medicine Centre, Institution Yenepoya Research Centre, Yenepoya University, University Road, Derlakatte, Mangalore 575018, Karnataka, India,*
E-mail:bipasha.bose@gmail.com

NMR Spectra of compounds IrL1-IrL5

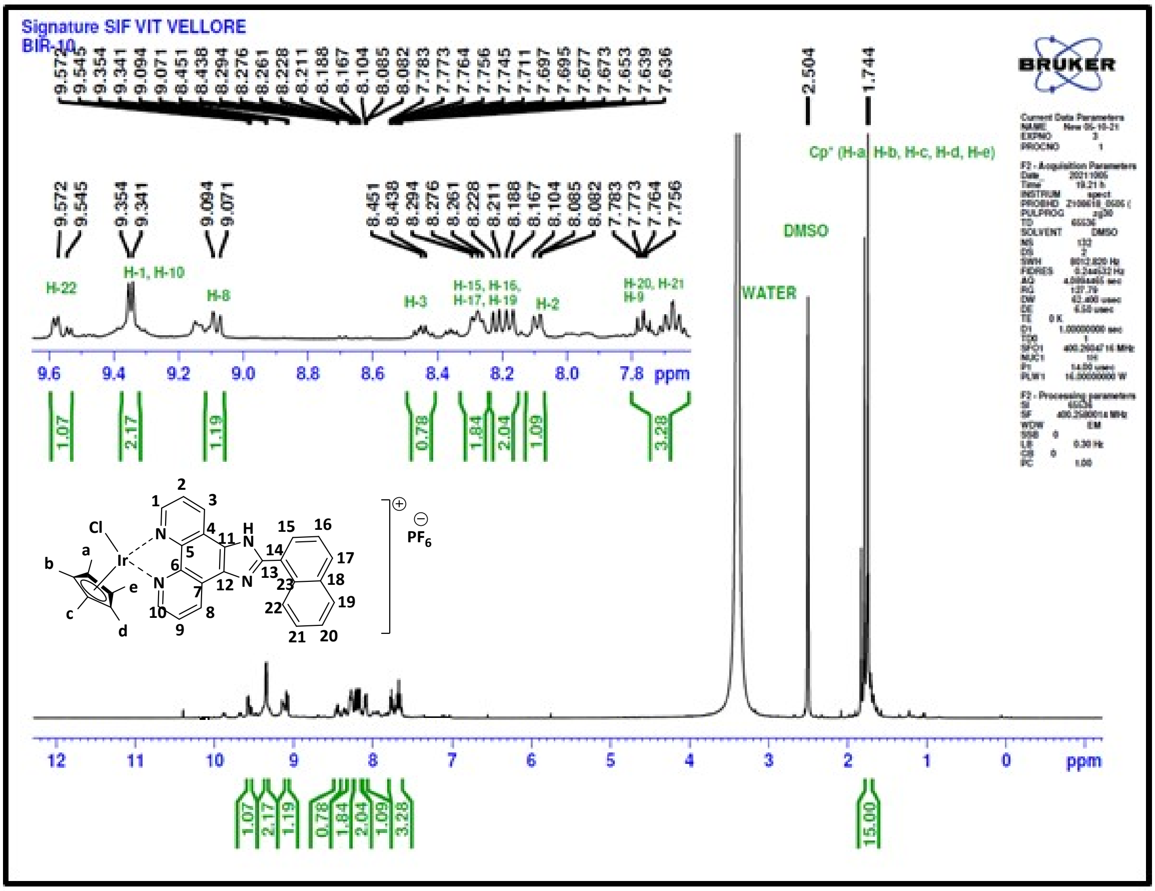

Figure S1- ^{1}H NMR of complex **IrL1**

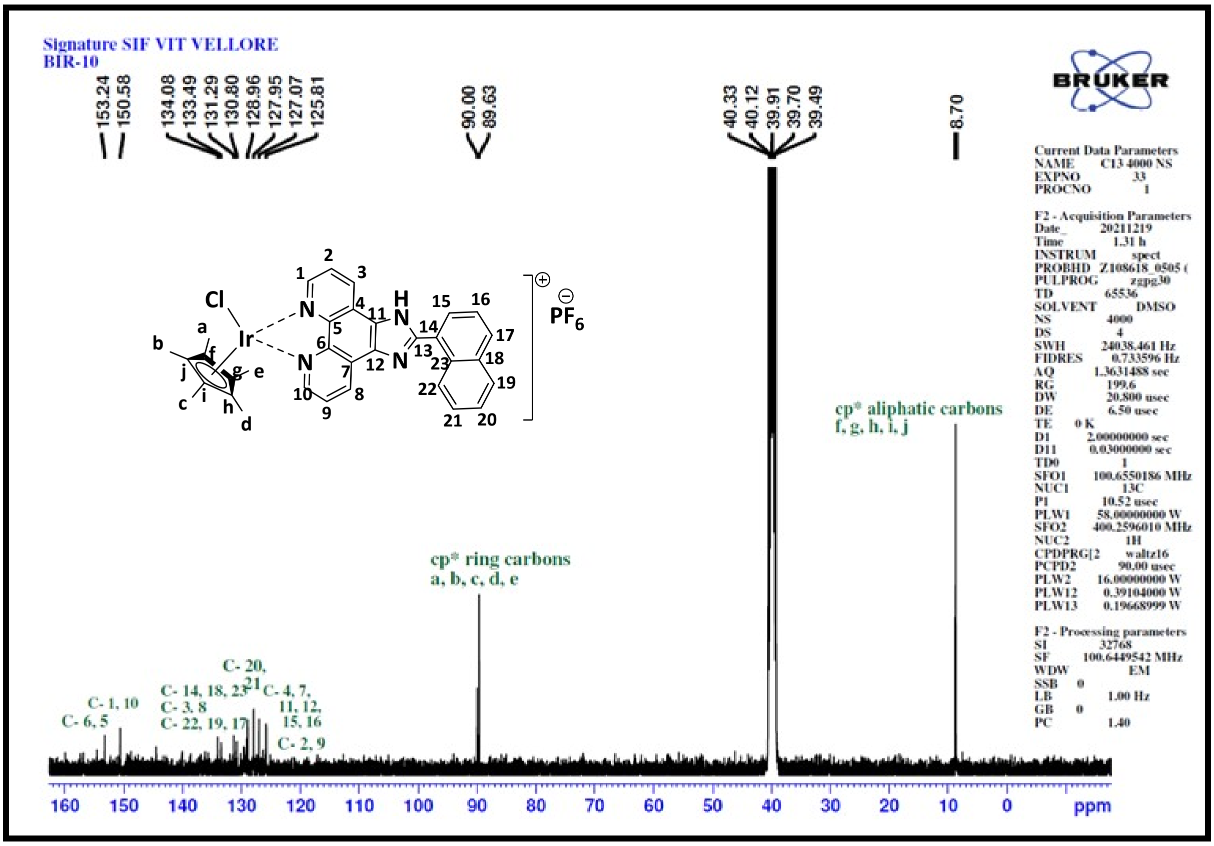

Figure S2- ^{13}C NMR of complex **IrL1**

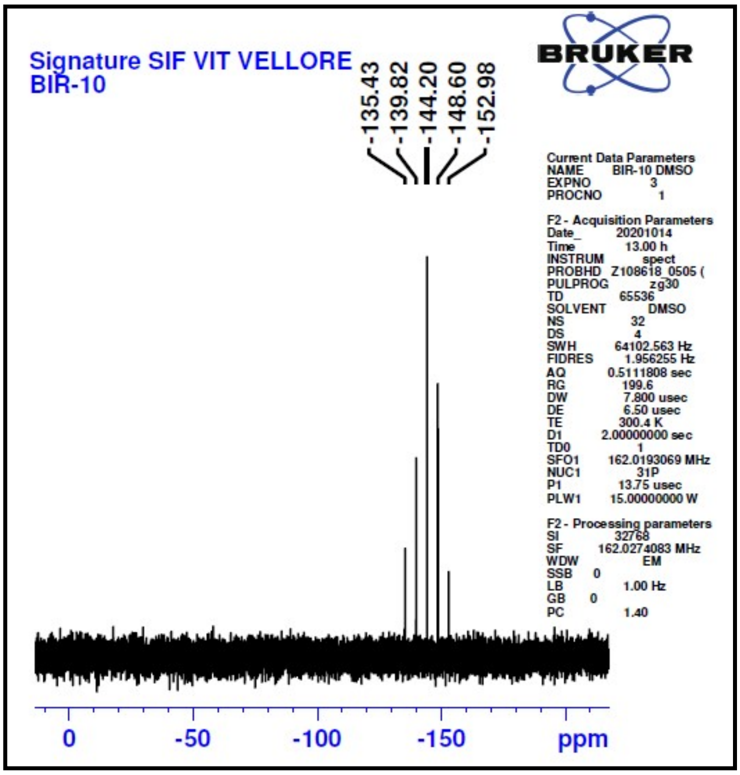

Figure S3- ^{31}P NMR of complex **IrL1**

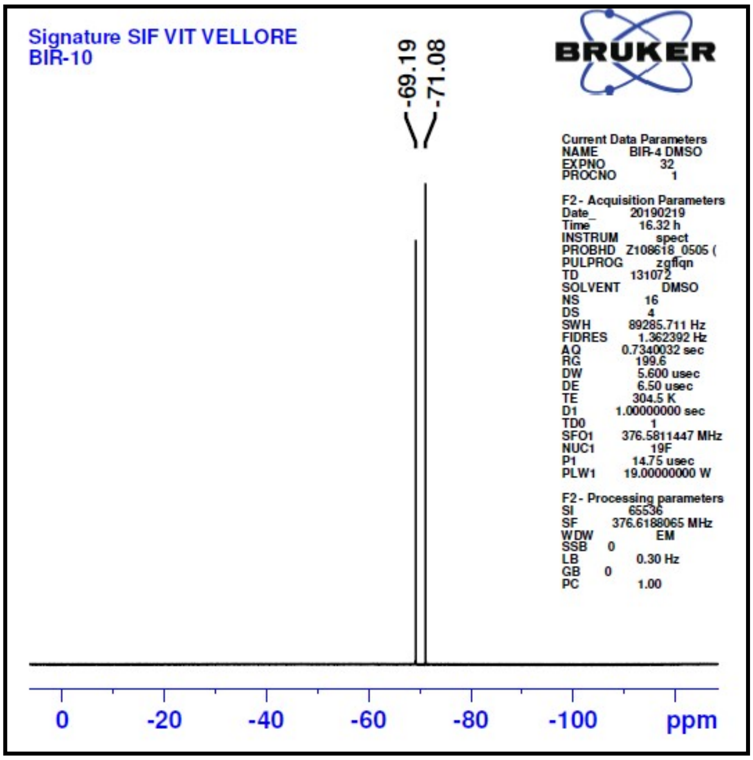

Figure S4- ^{19}F NMR of complex **IrL1**

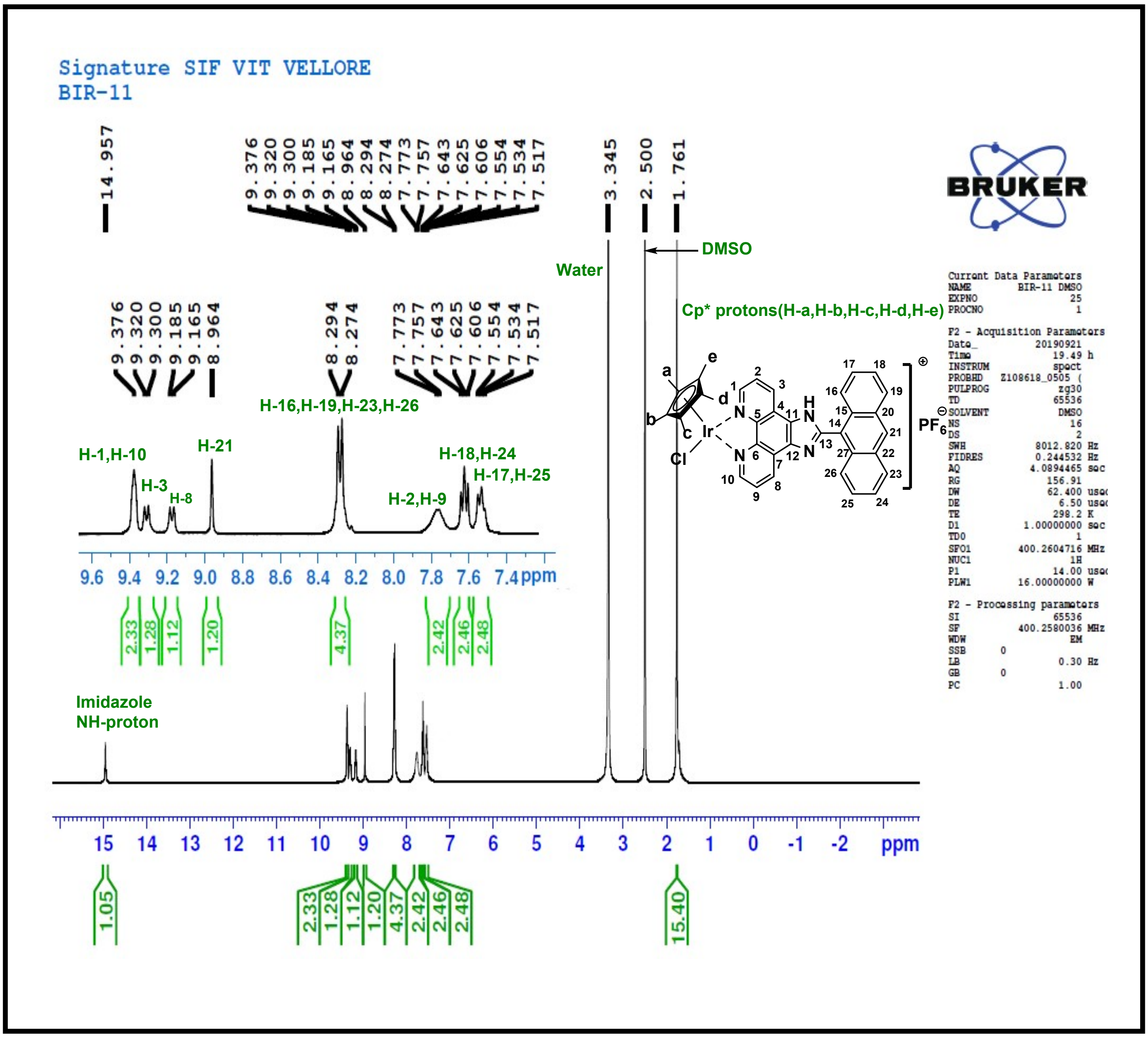

Figure S5- ^{1}H NMR of complex **IrL2**

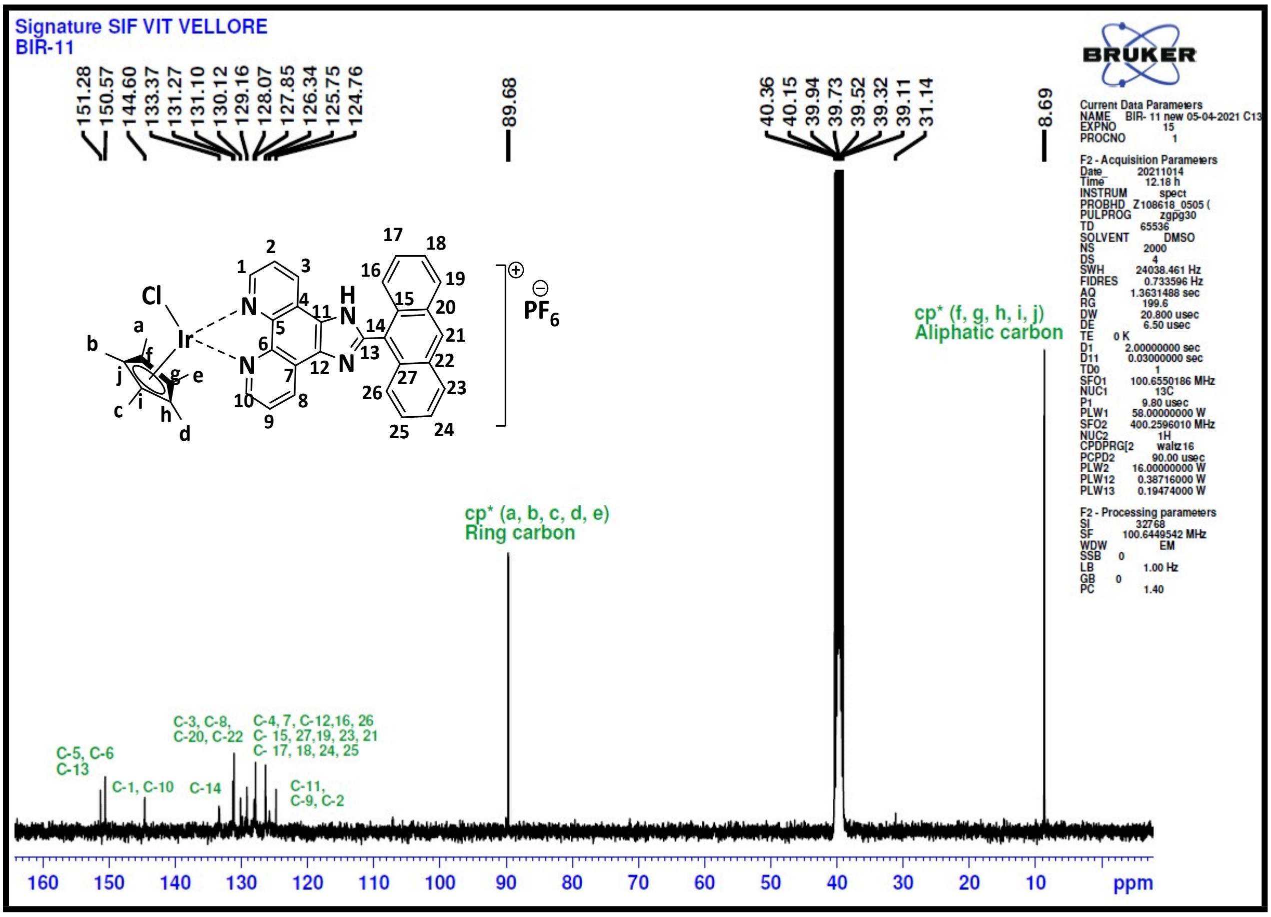

Figure S6- ^{13}C NMR of complex **IrL2**

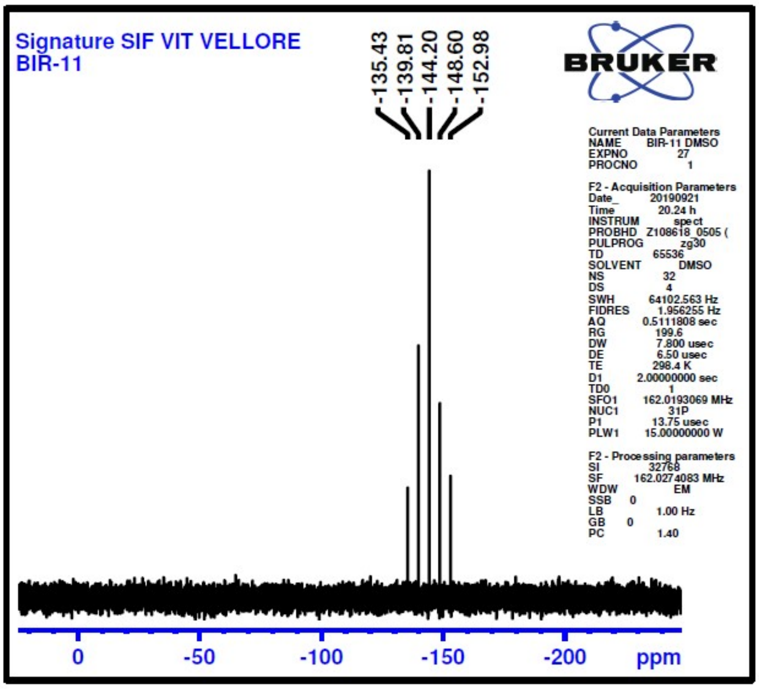

Figure S7- ^{31}P NMR of complex **IrL2**

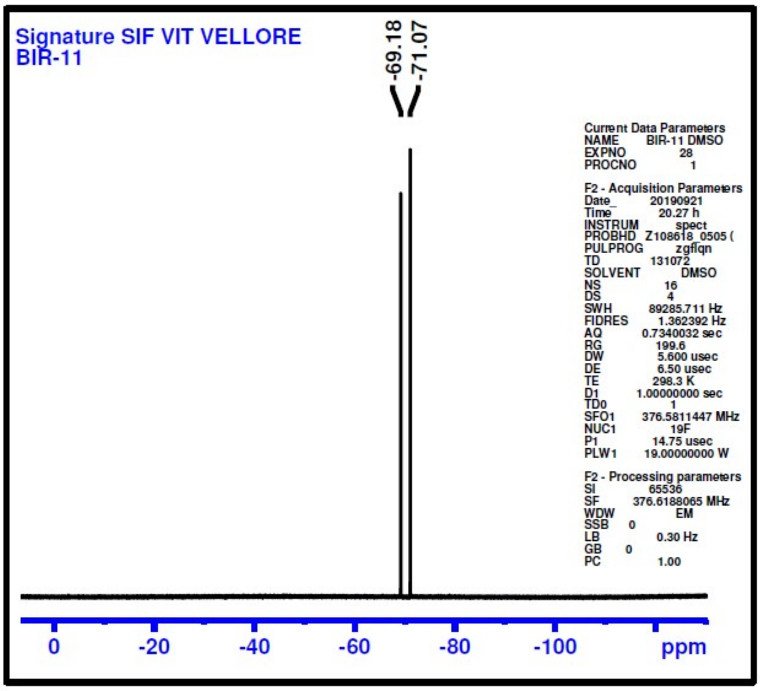

Figure S8- ^{19}F NMR of complex **IrL2**

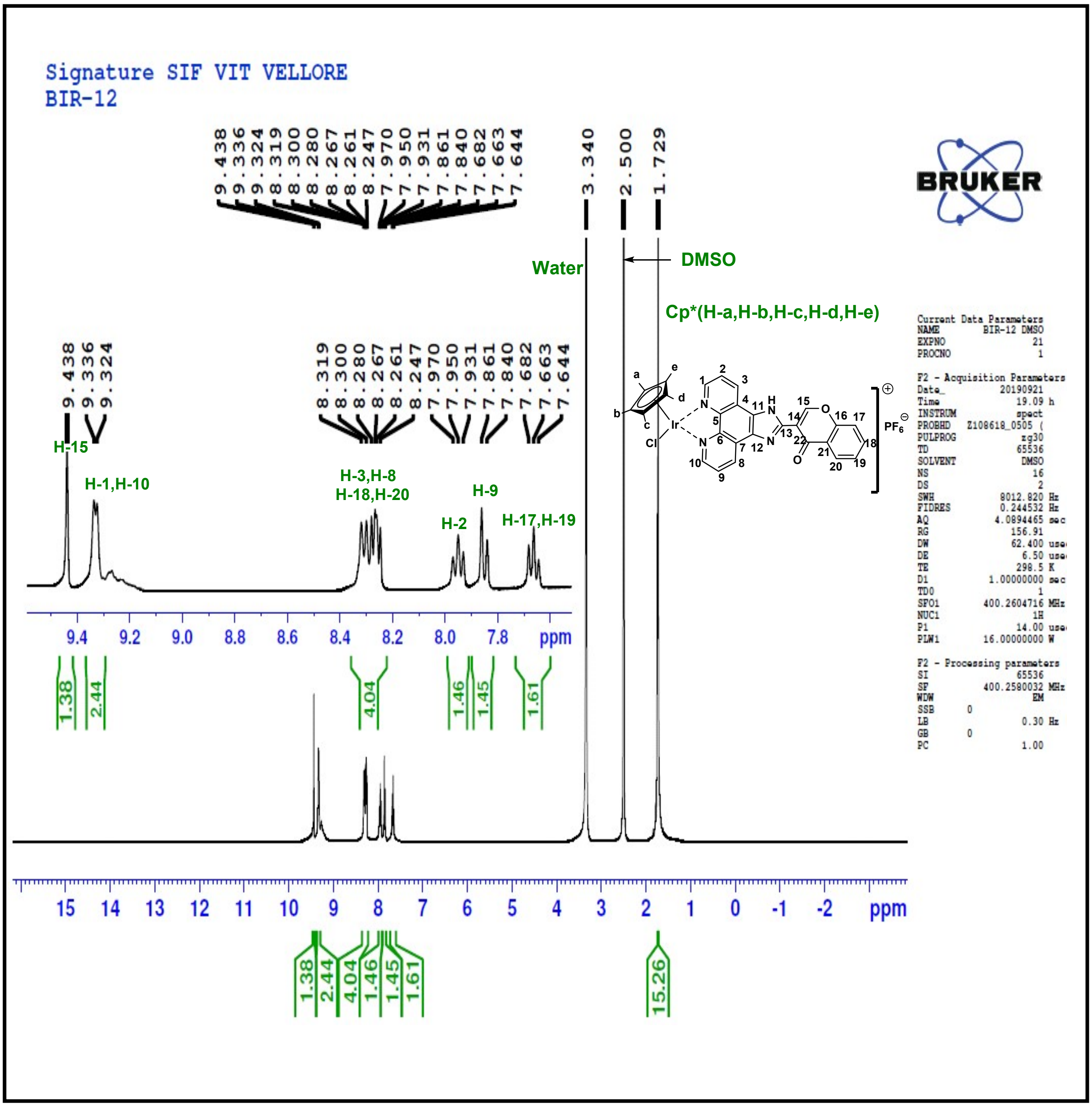

Figure S9- ^{1}H NMR of complex **IrL3**

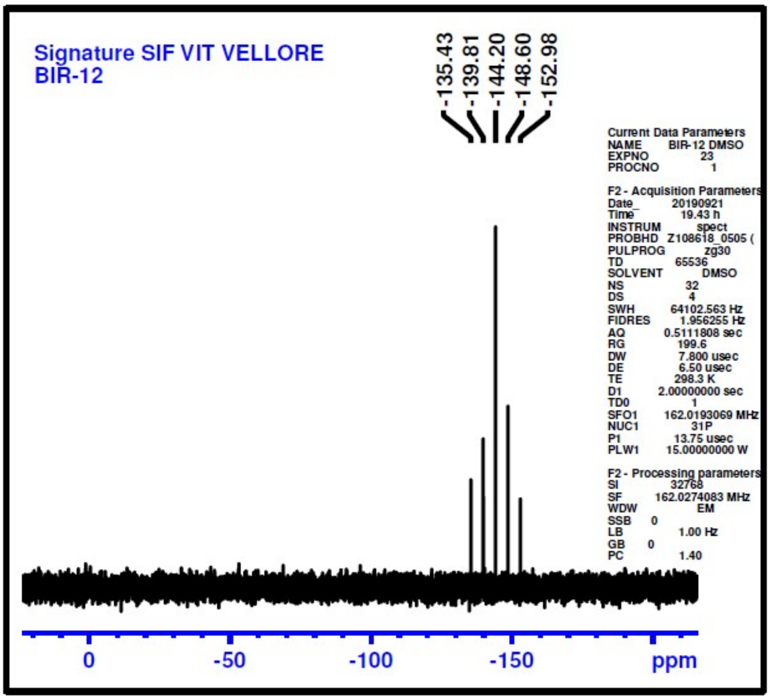

Figure S11- ^{31}P NMR of complex **IrL3**

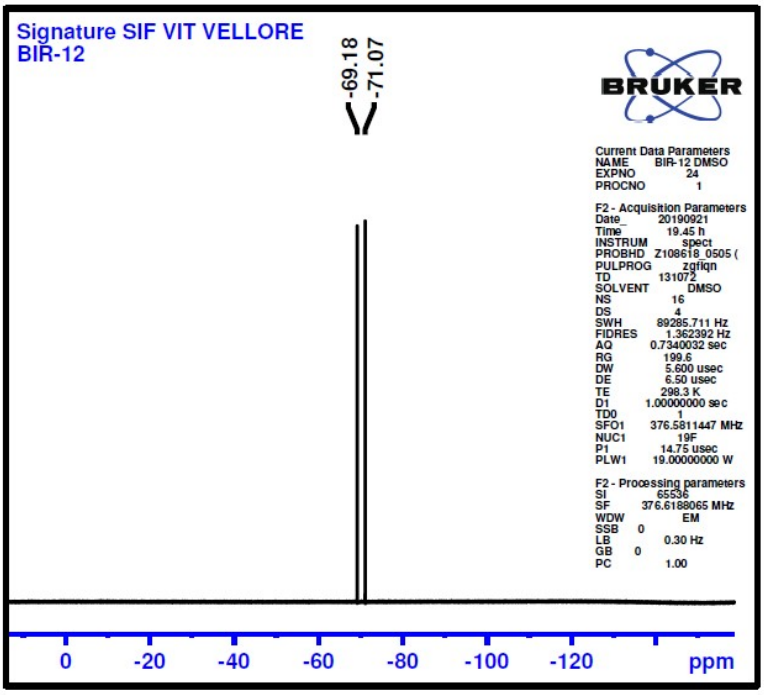

Figure S12- ^{19}F NMR of complex **IrL3**

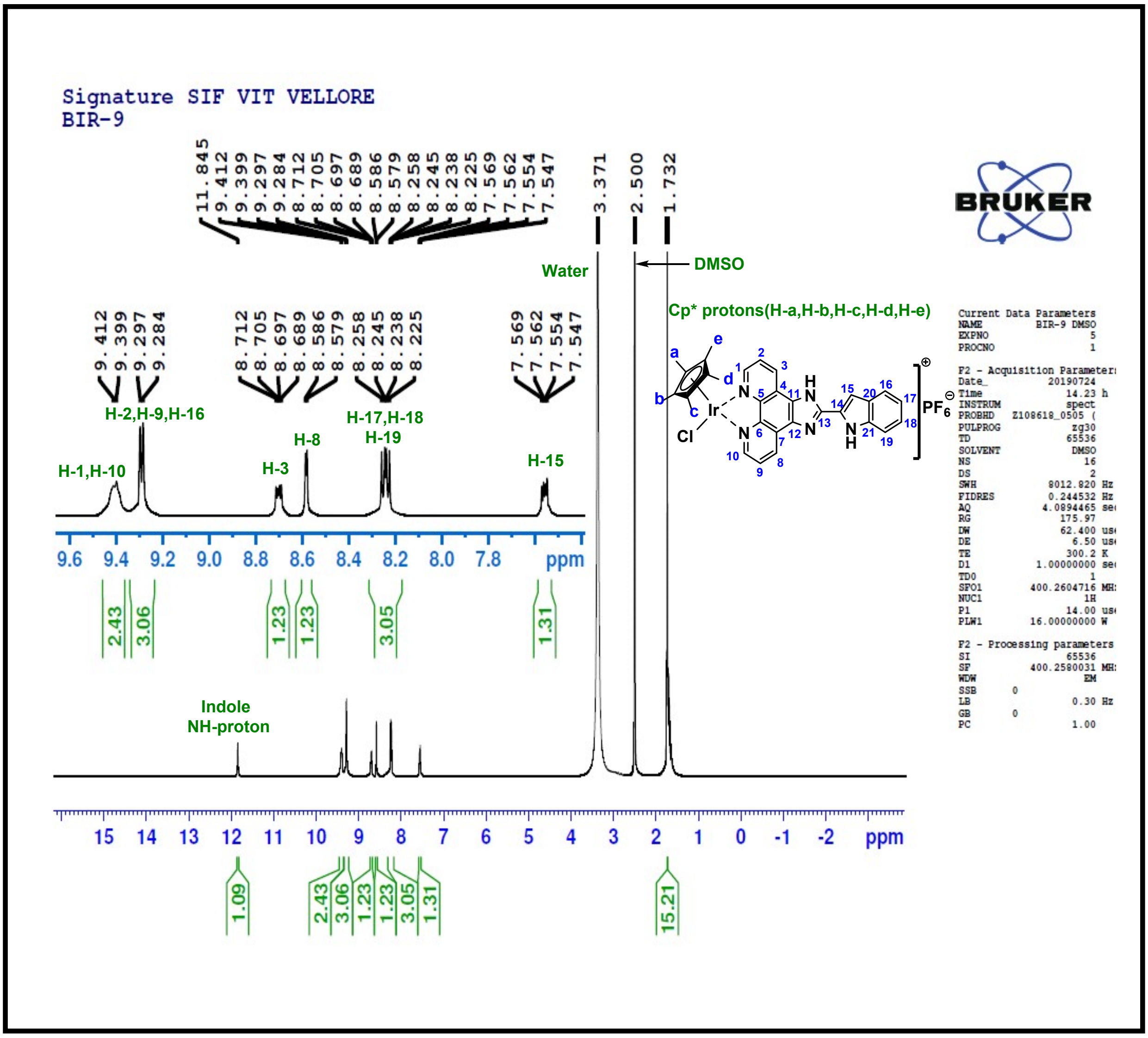

Figure S13- ^{1}H NMR of complex **IrL4**

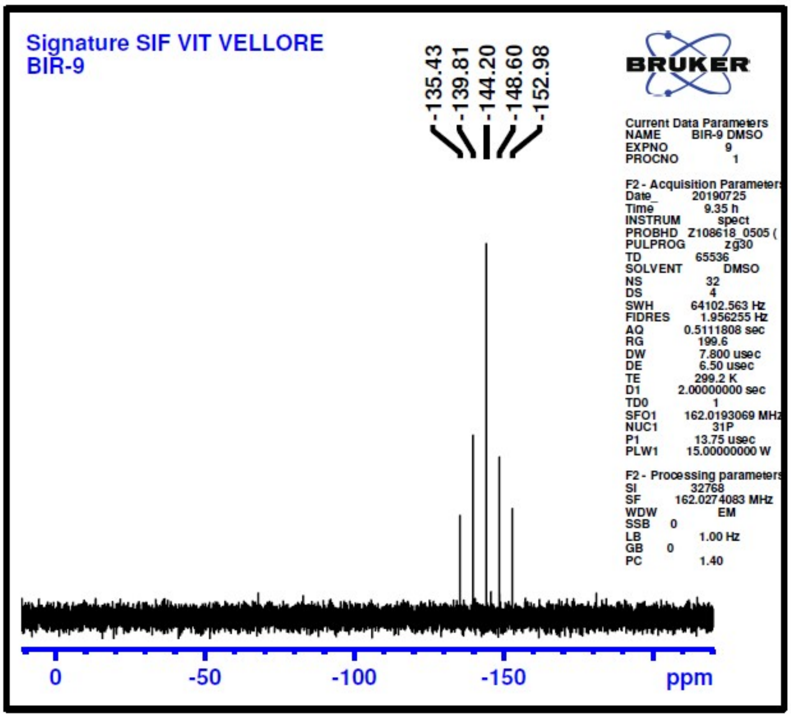

Figure S15- ^{31}P NMR of complex **IrL4**

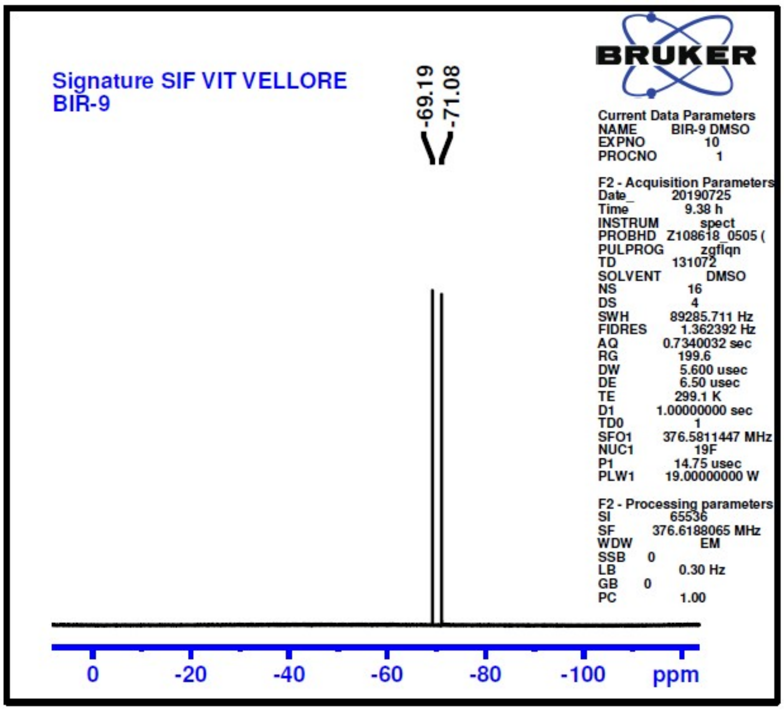

Figure S16- ^{31}P NMR of complex **IrL4**

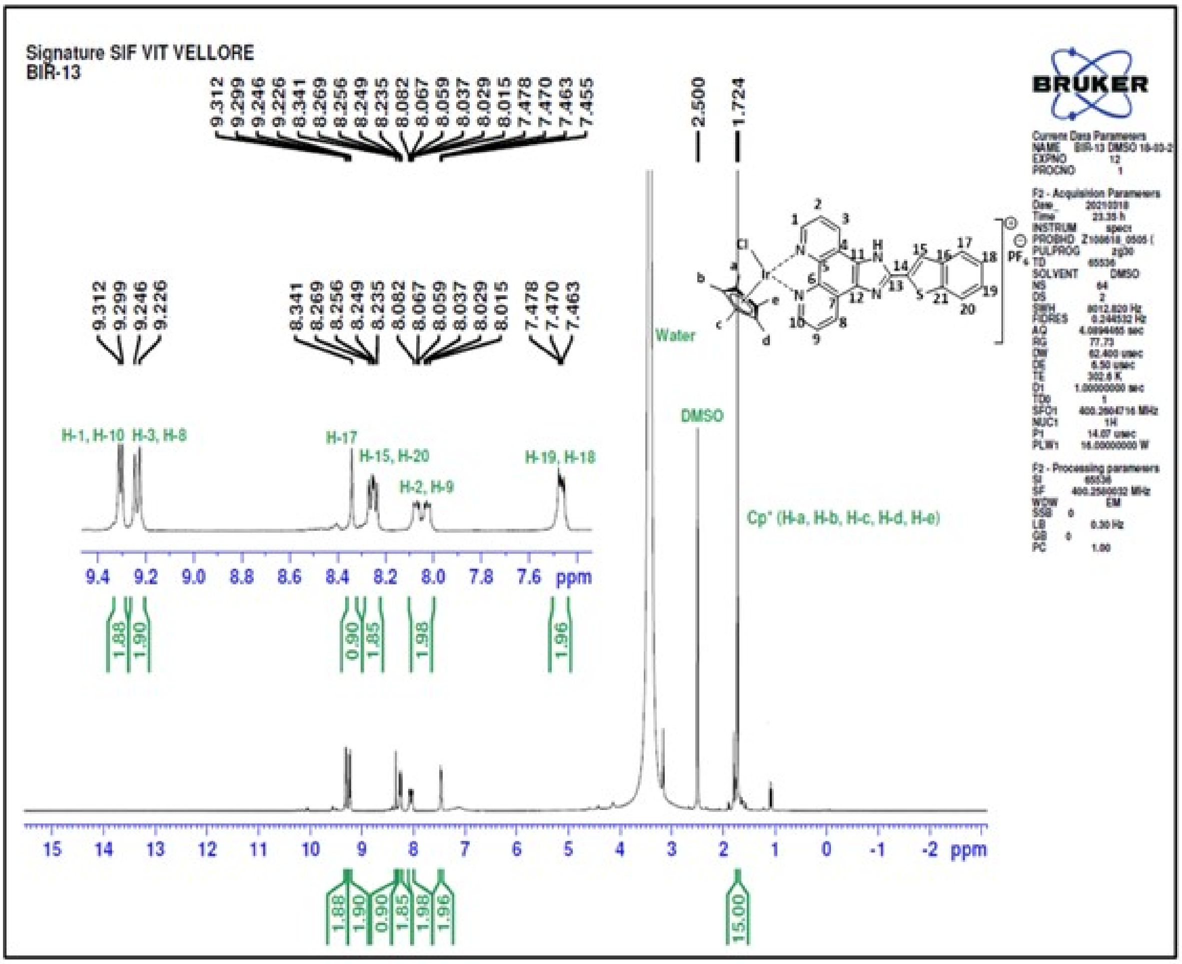

Figure S17- ^{1}H NMR of complex **IrL5**

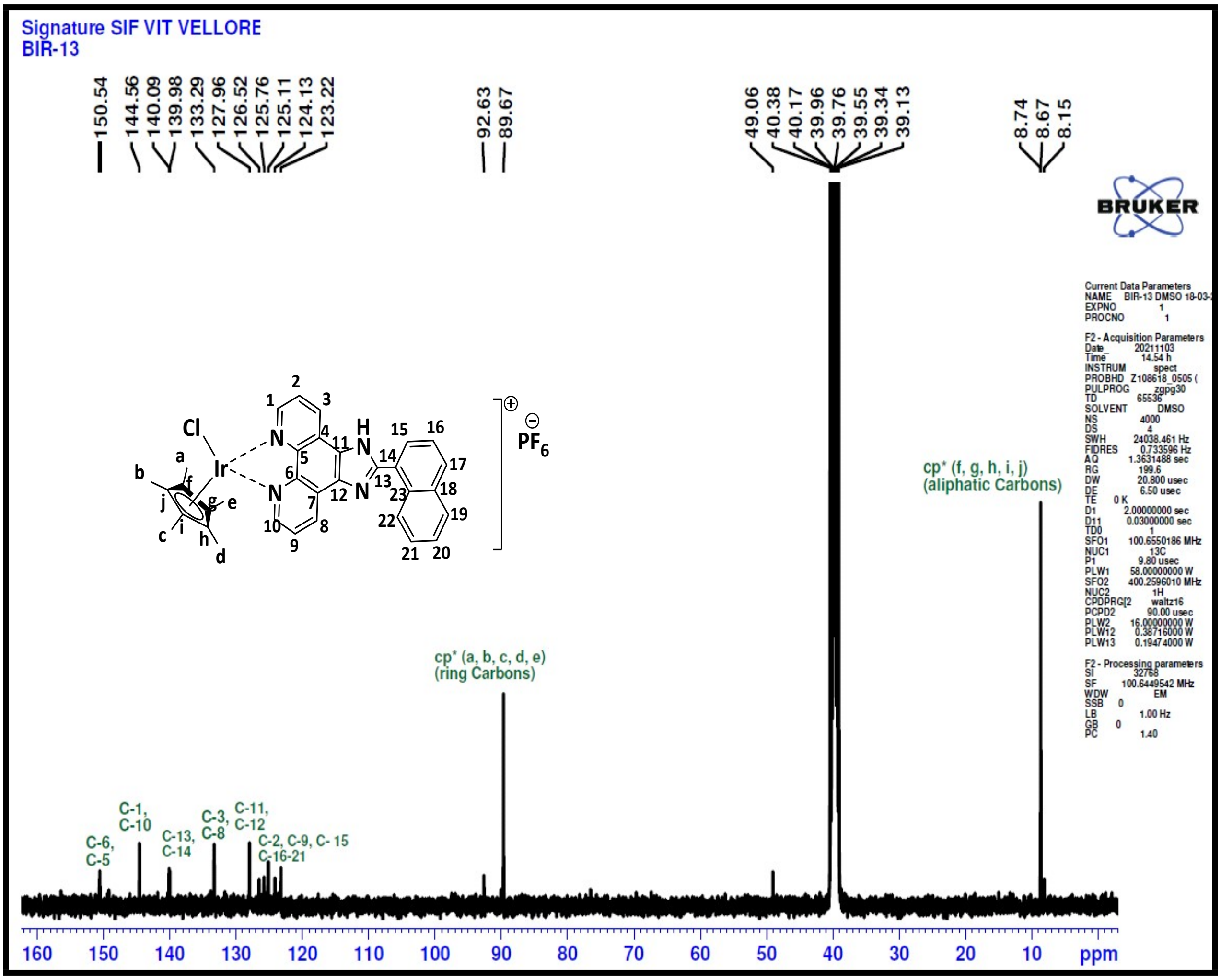

Figure S18- ^{13}C NMR of complex **IrL5**

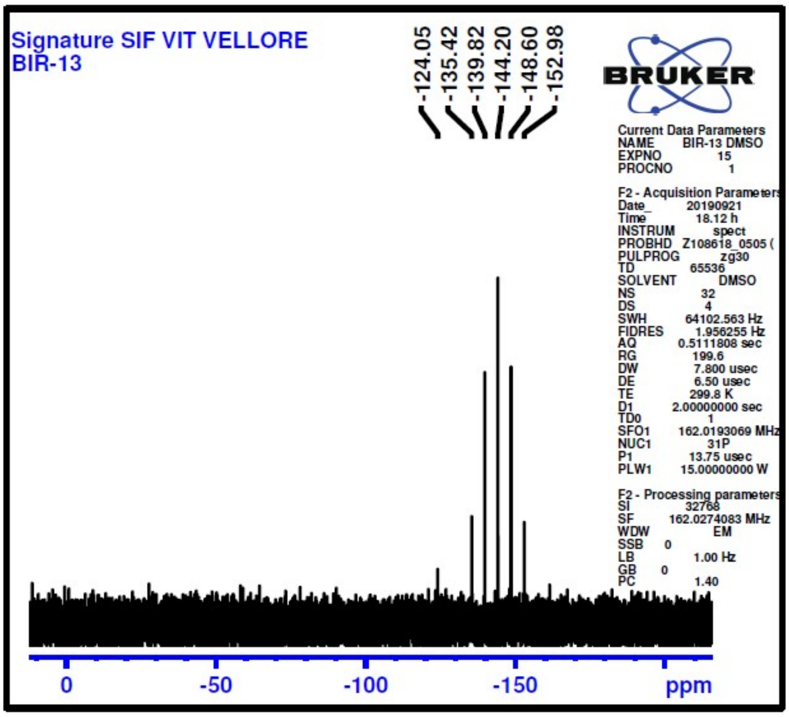

Figure S19- ^{31}P NMR of complex **IrL5**

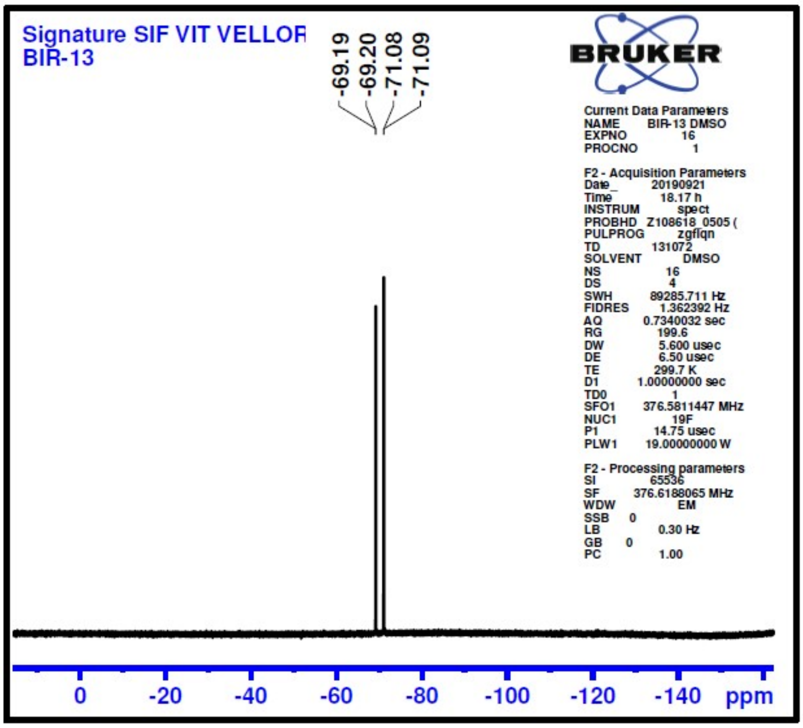

Figure S20- ^{19}F NMR of complex **IrL5**

Mass Spectra of compounds IrL1-5

TOTAL ION CHROMATOGRAM AND MOLECULAR ION (Q1) FOR 709.17 [M+]

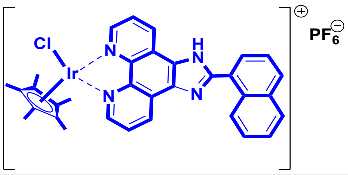

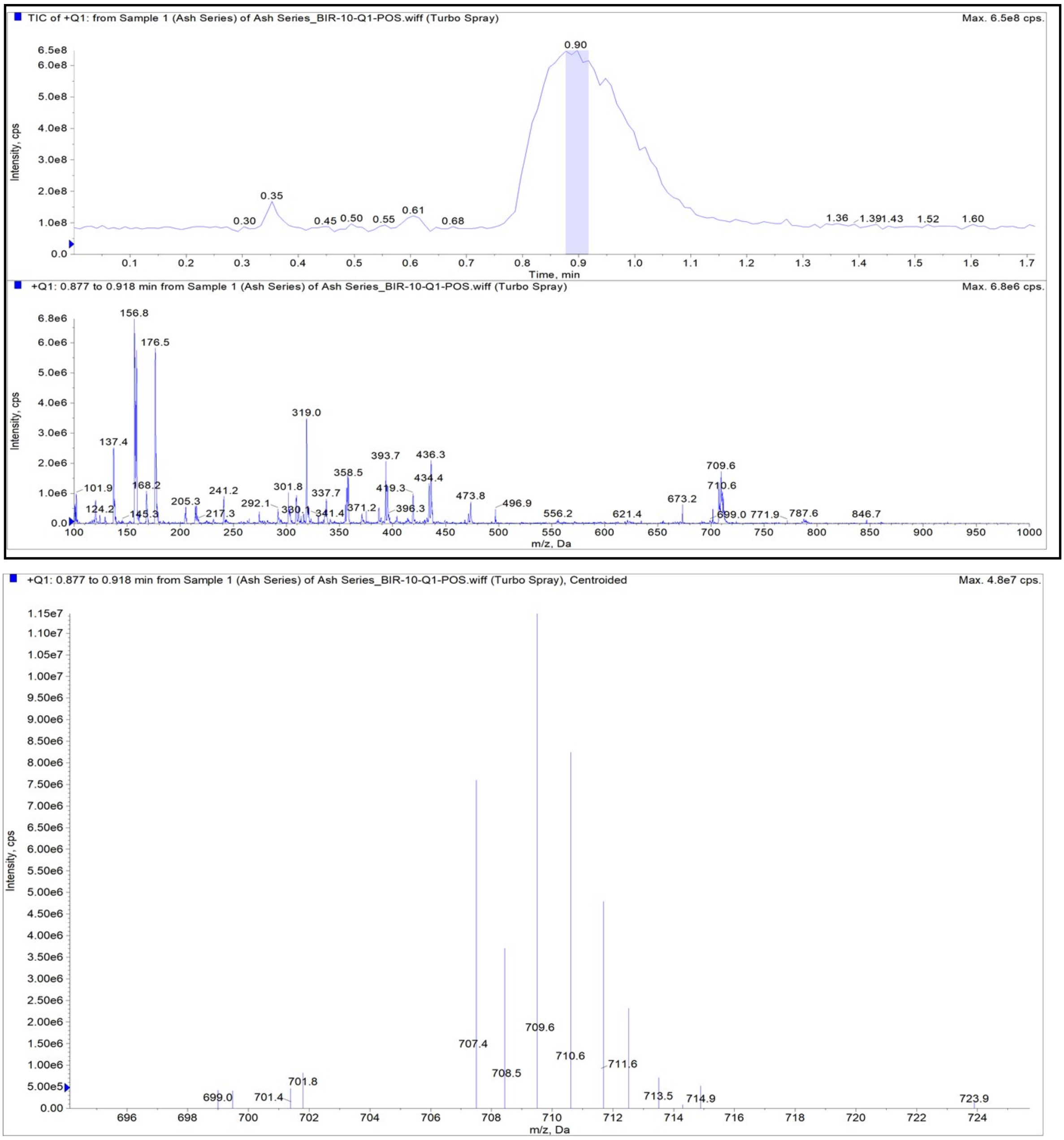

Figure S21- LC-MS spectra of complex **IrL1**

TOTAL ION CHROMATOGRAM AND MOLECULAR ION (Q1) FOR 759.19 [M$^+$]

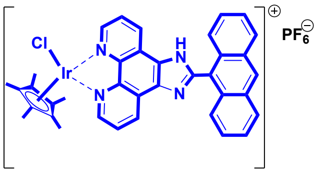

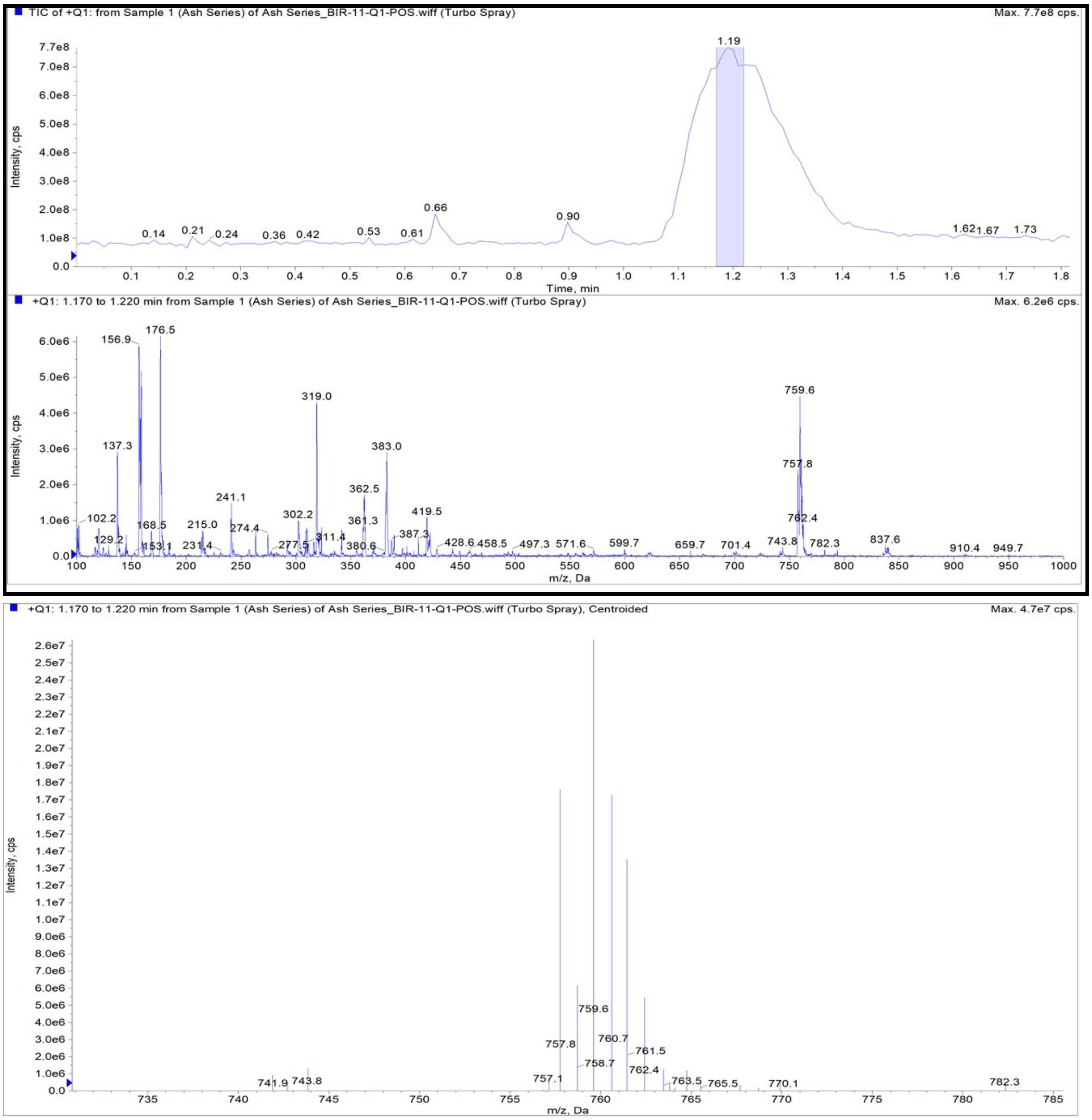

Figure S22- LC-MS spectra of complex **IrL2**

TOTAL ION CHROMATOGRAM AND MOLECULAR ION (Q1) FOR 698.17 [M⁺]

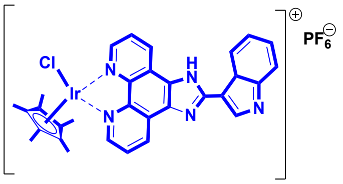

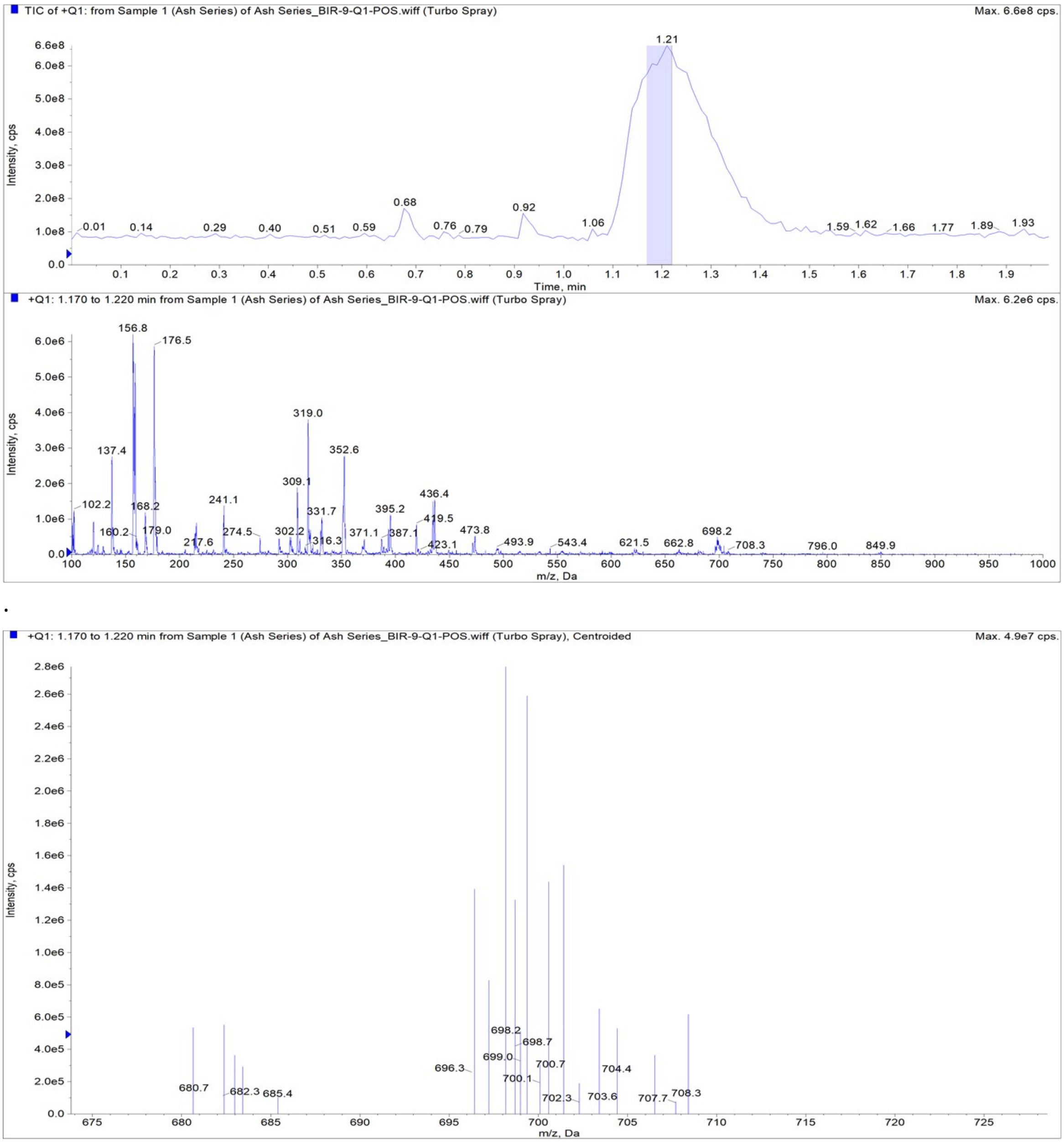

Figure S23- LC-MS spectra of complex **IrL4**

TOTAL ION CHROMATOGRAM AND MOLECULAR ION (Q1) FOR 715.13 [M+]

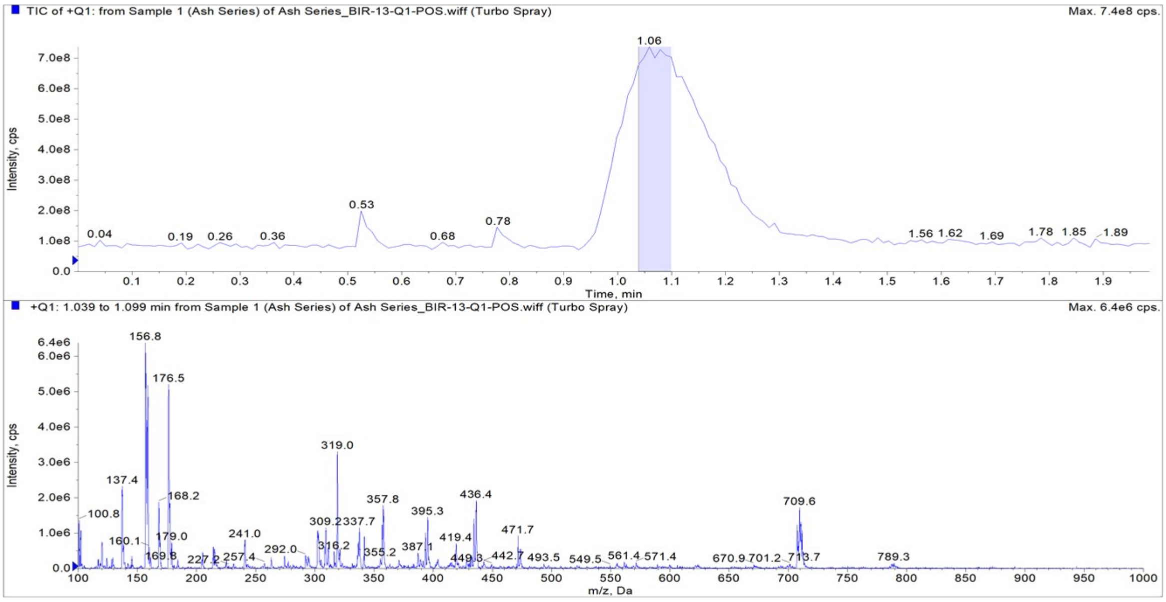

Figure S24- LC-MS spectra of complex **IrL5**

IR Spectra

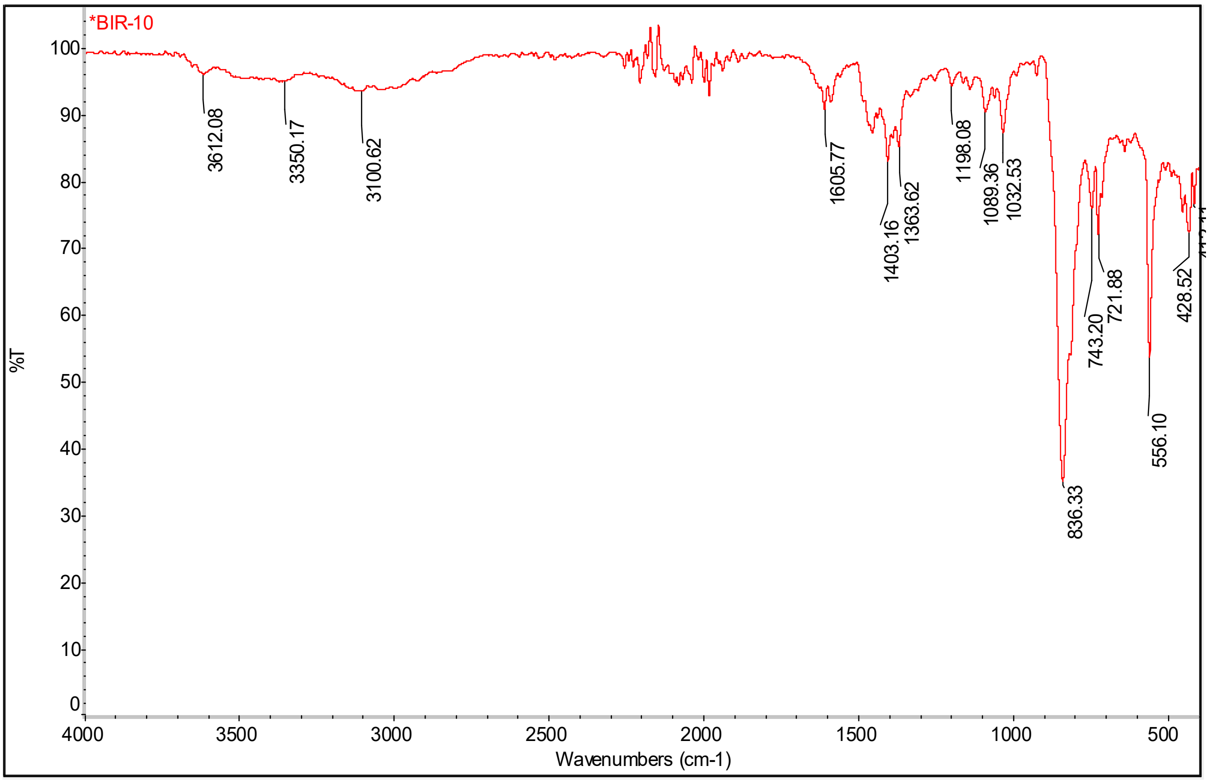

Figure S25- LC-MS spectra of complex **IrL1**

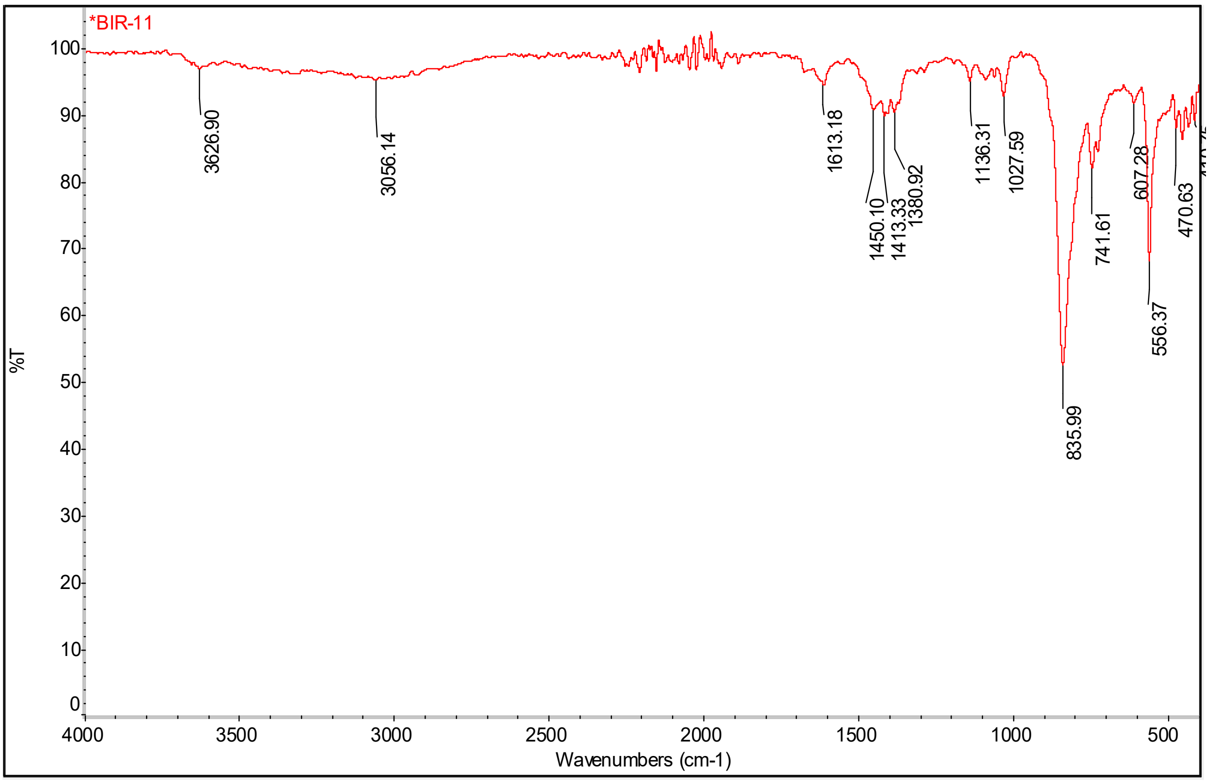

Figure S26- LC-MS spectra of complex **IrL2**

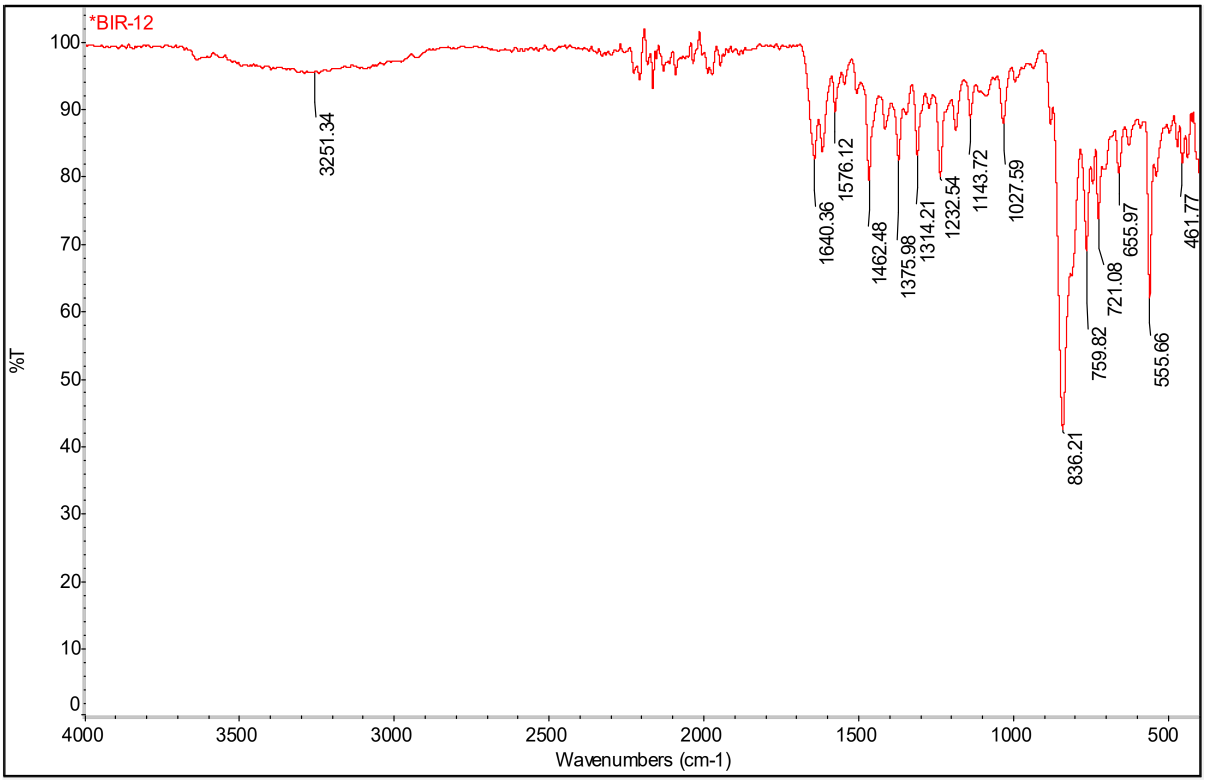

Figure S27- LC-MS spectra of complex **IrL3**

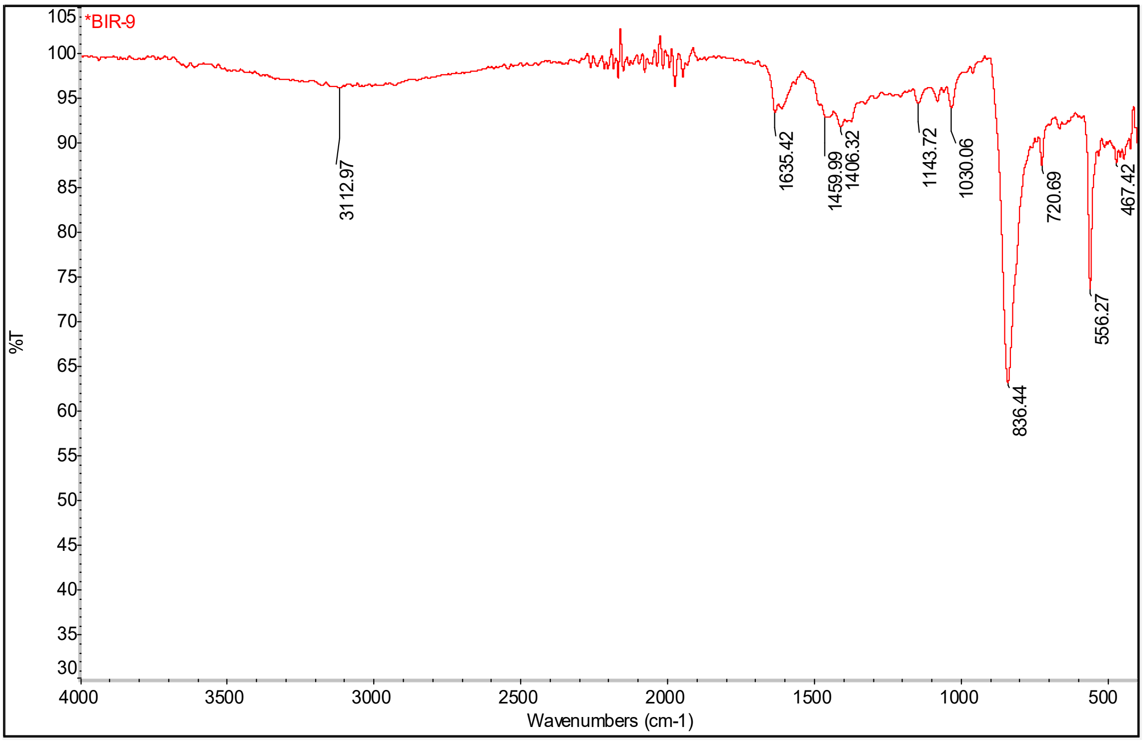

Figure S28- LC-MS spectra of complex IrL4

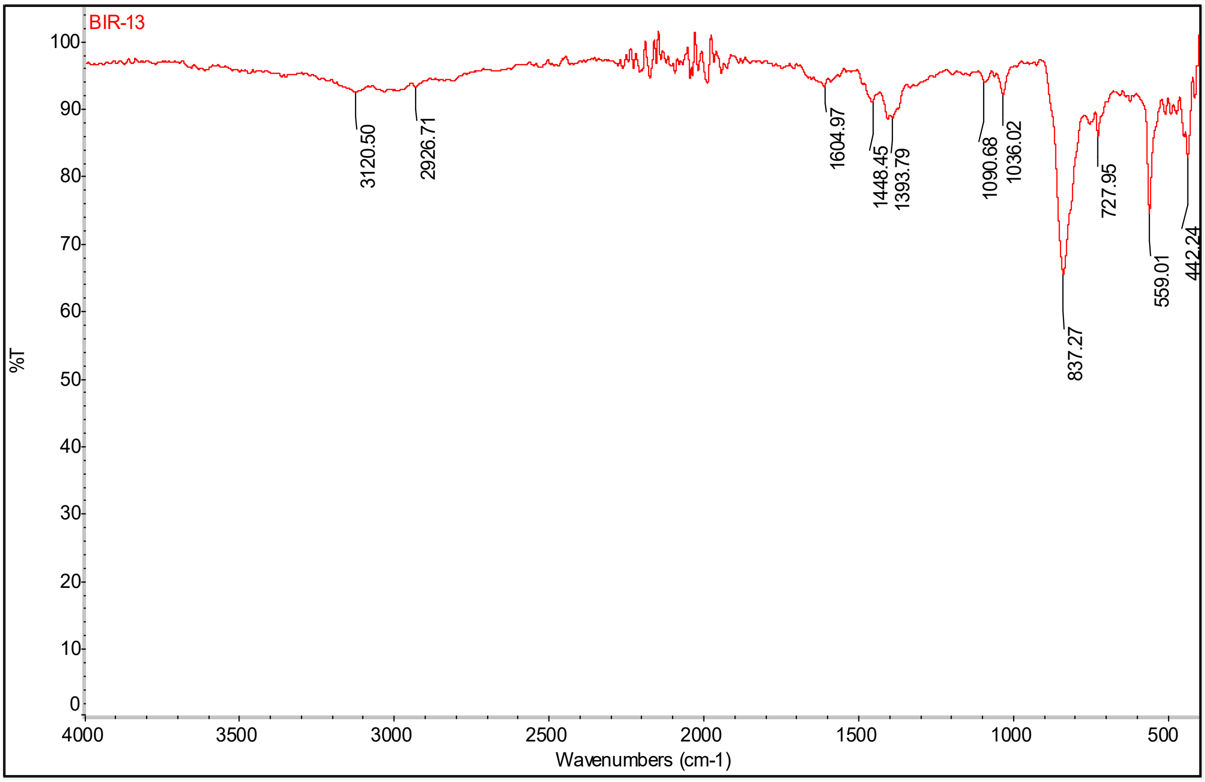

Figure S29- LC-MS spectra of complex IrL5

Experimental Section:

Biology Experiment

Cell culture:

For doing the cell culture the cells were retained in DMEM media (Gibco), added with 10% fetal bovine serum (Himedia, India), 1% penicillin and streptomycin and 1% of Glutmax (Gibco, Thermo Scientific, USA) at 37°C in 5% CO_2. When the cells attained 70%-80% confluency they were trypsinized using 0.25% trypsin-EDTA (Thermo Fisher Scientific, USA).

***In vitro* cytotoxicity**

In vitro cytotoxicity study was measured by standard MTT assay protocol.[1] First the prepared complexes (**IrL1-IrL5**) were dissolved in 0.1% DMSO followed by dilution with DMEM medium. One cancer cell lines *i.e.* triple negative breast cancer cells (MDA-MB-468) and one normal cell line i.e. immortalized human keratinocyte cell line (HaCaT) were used for this assay. Approximately 1×10^4 cells per well were cultured in 100 µl of a growth medium in 96-well plates and then incubated under 5% CO_2 atmosphere at 37°C temperature. Then the incubated cells were treated with different concentrations of the complexes in the volume of 100 µM/well. The cisplatin was taken as standard positive control for this experiment. The Cells which were in the control wells, also engaged the same volume of medium containing 0.1% DMSO. After 48 h, the medium was superfluous and cell cultures were again incubated with 100 µL of MTT reagent (1 mg/ml) for 5 h at 37° C. Then the resultant suspension was kept on micro vibrator for 10 min and the absorbance was recorded at λ = 570 nm in ELISA plate reader. The experiment was also performed in triplicate. The data were represented as the growth inhibition percentage i.e. % growth inhibition = 100 − [(AD × 100)/AB], where AD, measured absorbance in wells which contain samples and AB, measured absorbance for blank wells (cells with a medium and a vehicle).

Stability study

The stability studies of complex **IrL1** were conducted in six different solvents, i.e. aqueous DMSO (H_2O: DMSO = 9:1) and aqueous GSH medium respectively in presence or absence of different concentrations of NaCl.

DNA binding study

Electronic absorption spectroscopy was used to study the binding capacity of the complexes with calf-thymus DNA (Ct-DNA) and competitive binding assay as studied using ethidium bromide (EtBr) as quencher by fluorescence spectroscopy.

UV–visible studies[2]

DNA binding assay was carried out by using complexes **IrL5** in Tris-HCl buffer (5 mM Tris-HCl in water, pH 7.4) in aqueous medium. The concentration of Ct-DNA was calculated from its absorbance intensity at 260 nm and its known molar absorption coefficient value of 6600 $M^{-1}cm^{-1}$. Equal amount of DNA was added in both the sample and reference in cuvettes. Titration was carried out by increasing concentration of CT-DNA. On the eve of each measurement, sample was equilibrated with CT-DNA for about 5 min and then absorbance of the complex was measured. The intrinsic DNA binding constant (K_b) was calculated using the equation (i):

$$\frac{[DNA]}{(\varepsilon_a - \varepsilon_f)} = \frac{[DNA]}{(\varepsilon_b - \varepsilon_f)} + \frac{1}{K_b(\varepsilon_a - \varepsilon_f)} \text{L L} \quad (i)$$

Where [DNA] is the concentration of DNA in the base pairs, ε_a is the apparent extinction coefficient observed for the complex, ε_f corresponds to the extinction coefficient of the complex in its free form, and ε_b refers to the extinction coefficient of the complex when fully bound to DNA. The resultant data were plotted using Origin Lab, version 8.5 to obtain the [DNA]/(ε_a-ε_f) vs. [DNA] linear plot. The ratio of the slope to intercept from the linear fit gave the values of the intrinsic binding constants (K_b).

UV and Fluorescence study

UV and Fluorescence study of all these iridium (III) complexes, were executed in 10 % DMSO solution. Then the fluorescence quantum yields (Φ) were calculated by applying the comparative William's method which involves the use of well-characterized standard with known quantum yield value using 10% DMSO solution.[3] Quinine sulphate was used as a standard. Quantum yield was calculated according to the equation (ii):

$$\varphi = \varphi_R \times \frac{I_S}{I_R} \times \frac{OD_R}{OD_S} \times \frac{\eta_S}{\eta_R} \cdots\cdots\cdots (ii)$$

Where, φ = quantum yield, I = peak area, OD = absorbance at λmax, η = refractive index of solvent (s) and reference (R). Here, we have used quinine sulphate as a standard for calculating the quantum yield.

Ethidium bromide displacement assay

The ethidium bromide (EtBr) displacement assay was carried out to illustrate the mode of binding between the potent compounds with DNA.[4] The apparent binding constant (K_{app}) of the complexe **IrL1** to Ct-DNA were calculated using ethidium bromide (EtBr) as a spectral probe in 5 mM Tris-HCl buffer (pH 7.4). The values of the apparent binding constant (K_{app}) were obtained by using the equation (iii):

$$K_{app} \times [Complex]_{50} = k_{EtBr} \times [EtBr] \cdots\cdots\cdots (iii)$$

Where K_{EtBr} is the EtBr binding constant (K_{EtBr} = 1.0 x 10^7 M^{-1}), and [EtBr] = 8 x 10^{-6} M. Stern-Volmer equation was followed for quantitative determination of the Stern-Volmer quenching constant (K_{SV}).[5] Origin (8.5) software was used to plot the fluorescence data to obtain linear plot of I_0/I vs. [complex]. The value of K_{SV} was calculated from the following equation.

$$I_0/I = 1 + K_{SV}[Q] \cdots\cdots (iv)$$

Where I_0 = fluorescence intensity in absence of complex and I = fluorescence intensities in presence of complex of concentration [Q].

Protein binding studies

We are acquainted with the fact that serum albumin proteins are the main component. It is well known in blood plasma proteins and plays important roles in drug transport and metabolism, interaction of the drug with bovine serum albumin (BSA), a structural homologue of human serum albumin (HSA) was studied from tryptophan emission quenching experiment.[6] Tryptophan emission quenching experiment was performed to detect the interaction of the iridium complex **IrL1** with protein BSA. Initially, BSA solution (2 x 10^{-6} M) was prepared in Tris-HCl/NaCl buffer. The aqueous solutions of the complexes were subsequently added to BSA solution with gradual increase of their concentrations. After each addition, the solutions were shaken slowly for 5 min before recording the fluorescence at a wavelength of 295 nm (λex = 295 nm). A gradual decrease in fluorescence intensity of BSA at λ = 340 nm was observed upon increasing the concentration of complex, which confirmed

that the interaction between the complex and BSA was happened. Stern-Volmer equation was employed to quantitatively determine the quenching constant (K_{BSA}). Origin Lab, version 8.5 was used to plot the emission spectral data to obtain linear plot of I_0/I vs. [complex] using the equation (v) given below:

$$I_0/I = 1 + K_{BSA}[Q] = 1 + k_q\tau_0[Q] \ldots\ldots (v)$$

Where I_0 is the fluorescence intensity of BSA in absence of complex and I indicates the fluorescence intensity of BSA in presence of complex of concentration [Q], τ_0 = lifetime of the tryptophan in BSA found as 1 x 10^{-8} and kq is the quenching constant. Scatchard equation (vi) gives the binding properties of the complexes.[7] Where K = binding constant and n = number of binding sites.

$$\log(I_0 - I/I) = \log K + n\log[Q] \ldots\ldots (vi)$$

Conductivity measurement[8]

For authenticating the interaction of the complexes with DMSO and 10% aqueous DMSO, conductivity of the prepared complexes were performed using conductivity-TDS meter-307 (Systronics, India) and cell constant 1.0 cm^{-1}. Rate of conductivity was also estimated in different pH medium. Time dependent conductivity measurement was also carried out.

n-Octanol–water partition coefficient (log $P_{o/w}$)[9]

The log $P_{o/w}$ of the iridium complexes were adhering to shake flask method using the previously published procedure. A known amount of each complex (**IrL1-IrL5**) was suspended in water (pre-saturated with n-octanol) and shaken for 48 h on an orbital shaker. To allow the phase separation, the solution was centrifuged for 10 min at 3000 rpm. To obtain the partition coefficient, different ratios (0.5: 1, 1: 1, and 2: 1) of the saturated solutions were shaken with pre-saturated n-octanol for 20 min on an orbital shaker and followed the same procedure. Aliquots of the aqueous and octanol layers were pipetted out separately and the absorbances were measured with UV-Vis spectrophotometer using proper dilution. Each set was performed in triplicate, concentration of the substances in each layer was calculated using the respective molar extinction coefficients and the partition coefficient (log $P_{o/w}$) values were obtained from the ratio.

Notes and References

1. P. Liu, B. Wu, J. Liu, Y. Dai, Y. Wang, K. Wang, DNA Binding and Photocleavage Properties, Cellular Uptake and Localization, and in-Vitro Cytotoxicity of Dinuclear Ruthenium(II) Complexes with Varying Lengths in Bridging Alkyl Linkers, *Inorg. Chem.* **2016**, *55*, 1412−1422.
2. M. Sirajuddin, S. Ali, A. Badshah, Drug–DNA interactions and their study by UV–Visible, fluorescence spectroscopies and cyclic voltammetry, *J. Photochem. Photobio. B.*, **2013**, *124*, 1–19.
3. M. Shamsi-Sani, F. hirini, SM. Abedini, M. Seddighi, Synthesis of benzimidazole and quinoxaline derivatives using reusable sulfonated rice husk ash (RHA-SO3H) as a green and efficient solid acid catalyst, *Res. Chem. Intermed.*, **2016**, *42*, 1091–1099.
4. S. Dasari, A. K. Patra, Luminescent europium and terbium complexes of dipyridoquinoxaline and dipyridophenazine ligands as photosensitizing antennae: structures and biological perspectives, *Dalton Trans.*, **2015**, *44*, 19844-19855.
5. J. Keizer, Nonlinear fluorescence quenching and the origin of positive curvature in Stern-Volmer plots, *J. Am. Chem. Soc.*, **1983**, *105*, 1494–1498.
6. V. D. Suryawanshi, L. S. Walekar, A. H. Gore, P. V. Anbhule, G. B. Kolekar, Spectroscopic analysis on the binding interaction of biologically active pyrimidine derivative with bovine serum albumin, *J. Pharm. Anal.*, **2016**, *6*, 56–63.
7. K. Jeyalakshmi, J. Haribabu, C. Balachandran, S. Swaminathan, N. S. P. Bhuvanesh, R. Karvembu, Coordination Behavior of N,N′,N″-Trisubstituted Guanidine Ligands in Their Ru–Arene Complexes: Synthetic, DNA/Protein Binding, and Cytotoxic Studies, *Organometallics,* **2019**, *38*, 753−770.
8. S. Nikolić, L. Rangasamy, N. Gligorijević, S. Aranđelović, S. Radulović, G. Gasser, S. Grgurić-Šipka, Synthesis, characterization and biological evaluation of novel Ru(II)–arene complexes containing intercalating ligands, *J. Inorg. Biochem.* **2016**, *160*, 156–165.
9. M. Kubanik, H. Holtkamp, T. Söhnel, S. M. F. Jamieson, C. G. Hartinger, Impact of the Halogen Substitution Pattern on the Biological Activity of Organoruthenium 8-Hydroxyquinoline Anticancer Agents, *Organometallics*, **2015**, *34*, 5658–5668.
